# The Skeleton Forming Proteome of an Early Branching Metazoan: A Molecular Survey of the Biomineralization Components Employed by the Coralline Sponge *Vaceletia* Sp.

**DOI:** 10.1371/journal.pone.0140100

**Published:** 2015-11-04

**Authors:** Juliane Germer, Karlheinz Mann, Gert Wörheide, Daniel John Jackson

**Affiliations:** 1 Department of Geobiology, Georg-August University of Göttingen, Göttingen, Germany; 2 Max Planck Institute of Biochemistry, Department of Proteomics and Signal Transduction, Munich, Germany; 3 Department of Earth- and Environmental Sciences & GeoBio-Center, Ludwig-Maximilians-Universität München, München, Germany; 4 SNSB—Bavarian State Collections of Palaeontology & Geology, München, Germany; Russian Academy of Sciences, Institute for Biological Instrumentation, RUSSIAN FEDERATION

## Abstract

The ability to construct a mineralized skeleton was a major innovation for the Metazoa during their evolution in the late Precambrian/early Cambrian. Porifera (sponges) hold an informative position for efforts aimed at unraveling the origins of this ability because they are widely regarded to be the earliest branching metazoans, and are among the first multi-cellular animals to display the ability to biomineralize in the fossil record. Very few biomineralization associated proteins have been identified in sponges so far, with no transcriptome or proteome scale surveys yet available. In order to understand what genetic repertoire may have been present in the last common ancestor of the Metazoa (LCAM), and that may have contributed to the evolution of the ability to biocalcify, we have studied the skeletal proteome of the coralline demosponge *Vaceletia* sp. and compare this to other metazoan biomineralizing proteomes. We bring some spatial resolution to this analysis by dividing *Vaceletia’s* aragonitic calcium carbonate skeleton into “head” and “stalk” regions. With our approach we were able to identify 40 proteins from both the head and stalk regions, with many of these sharing some similarity to previously identified gene products from other organisms. Among these proteins are known biomineralization compounds, such as carbonic anhydrase, spherulin, extracellular matrix proteins and very acidic proteins. This report provides the first proteome scale analysis of a calcified poriferan skeletal proteome, and its composition clearly demonstrates that the LCAM contributed several key enzymes and matrix proteins to its descendants that supported the metazoan ability to biocalcify. However, lineage specific evolution is also likely to have contributed significantly to the ability of disparate metazoan lineages to biocalcify.

## Introduction

Biomineralization is a phenomenon that can be found throughout the tree of life. Its appearance in the metazoan (animal) fossil record coincides with a rapid increase in their morphological diversity, suggesting that the evolution of this ability was one key factor that supported the Cambrian explosion (~540 mya). Much effort has therefore been aimed at elucidating the genetic and molecular mechanisms that underlie the ability to biomineralize in disparate animal phyla. It has been proposed that the metazoan ability to build mineral skeletal elements evolved at least twenty times independently [1]. However, this estimate makes assumptions regarding the morphological homology of skeletal elements in disparate taxa, and assumes simplistic models of evolutionary gain/loss of mineralized elements while disregarding the underlying molecular mechanisms that fabricate these structures. Available skeletal proteome datasets from metazoans such as molluscs [2–5], sea urchins [6, 7] and brachiopods [8–10] go some way towards addressing this issue, but to study the origins of metazoan biomineralization it is crucial to investigate the biomineralizing proteome of an early branching metazoan.

Sponges (Phylum Porifera) have traditionally been considered to be the earliest branching surviving metazoan lineage (reviewed in [11, 12]). However, resolving deep metazoan relationships, especially those among the non-bilaterian taxa Porifera, Ctenophora, Cnidaria and Placozoa, is still a challenging task (see [13]) and the branching order close to the root of the animal Tree of Life is not unequivocally accepted. Recent studies using molecular phylogenetic analyses, transcriptomic and genomic data either confirm [14–16] or reject [17–20] the view of sponges as the sister group to all other animals. Resolving the phylogeny of the non-bilaterian phyla is crucial to understand the evolution of metazoan traits such as epithelia, nerves and muscles, as well as biomineralization.

Despite the ongoing discussions about their placement in the metazoan tree, sponges are among the first animals represented in the fossil record to display a “biologically controlled” mode of biomineralization [1]. During the Tommotian Age (beginning 530 MYA) the Archaeocyatha, an assemblage of organisms which most authorities now agree were an extinct class of sponges [21], began to leave evidence in the fossil record of a mode of heavy calcification that is poorly represented among living sponges. As the planet’s first metazoan reef builders, Archaeocyathids were ecologically important, globally distributed, and were taxonomically diverse with hundreds of recognized species [22–24]. The Archaeocyathids have been extinct since the Cambrian, however superficial similarities in some skeletal features of these ancient animals have been described from a single living (‘sphinctozoan’-like) taxon *Vaceletia*, which first appears in the Middle Triassic [25]. Based on these superficial morphological similarities some authors have argued that *Vaceletia* may be a modern Archaeocyath [25, 26]. However, this is very likely not the case since molecular data has shown that the genus *Vaceletia* belongs to the Dictyoceratida within the Class Demospongiae [27–29]. Nonetheless, taxon *Vaceletia* represents an early branching metazoan with a possible ancient mode of biomineralization and this makes it an ideal candidate to deepen our understanding of how the ability to biomineralize may have first arisen in sponges. Taxon *Vaceletia* has been regarded as a monospecific genus with the single type species *Vaceletia crypta* [30]. However, several different growth forms have been discovered in the last decades (see discussion in [27]) and their taxonomy is not fully resolved yet. In this present study we obtained data from a yet to be described likely new colonial-branching *Vaceletia* species from Osprey- and Bougainville Reefs [27, 31] (Coral Sea, Australia).

**Fig 1:**
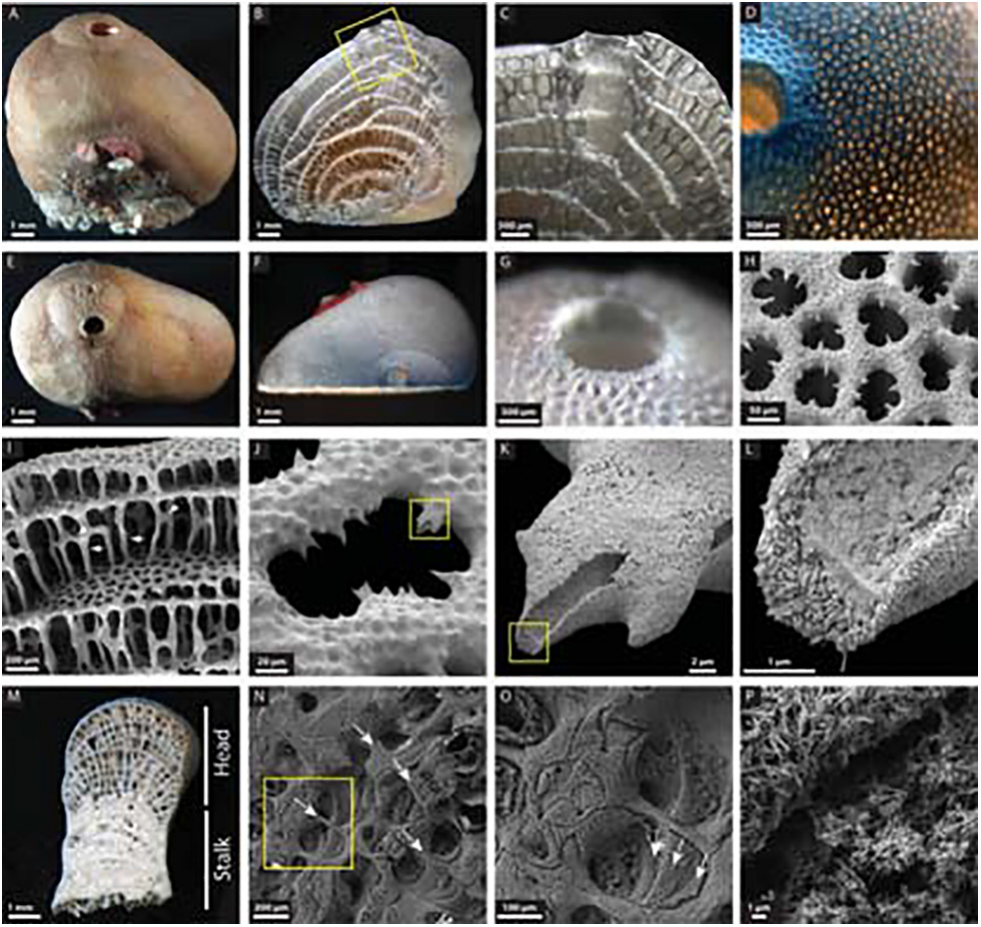
General morphological features of *Vaceletia* sp. and its CaCO_3_ skeleton. (A) A lateral view of a fixed animal. The exhalent siphon (arrow) is clearly visible. (B) A sagittal section view after treatment with NaOCl and grinding to reveal the interior structure of the skeleton. (C) Magnification of the boxed section in B illustrates the structure of the siphon and pillars which support each terraced chamber. The ontogenetically youngest chamber is at the top of the animal (arrow). (D) An apical view with transmitted light through the specimen following NaOCl treatment to highlight the elaborate structure of the ostia. (E) An apical view of the intact animal. (F) An apical view of the animal following treatment with NaOCl and grinding to the sagittal plane. (G) Magnified view of the siphon following treatment with NaOCl. (H) SEM image of the ostia illustrating the unique pattern of inward facing spines. (I) SEM image roughly equivalent to the boxed section in B. Pillars (arrows) support each chamber, and are reinforced by radial spines (arrowhead). (J) Magnification of an ostium. (K) Magnification of the damaged inward facing spine boxed in J. (L) Magnification of the tip of the damaged spine boxed in K. Individual crystals of aragonite are clearly visible. (M) A sagittal section view after treatment with NaOCl and grinding shows the head and hypercalcified stalk regions. (N) SEM image of the stalk region after being etched with EDTA. Note that the pillars of the skeleton are still visible (arrows). (O) Magnification of the boxed section in N shows that the chambers are mineralized in layers (arrows). Note that not all chambers are mineralized entirely. (P) Both pillars and mineralized chambers are constructed by needles of aragonite.

At length scales ranging from the cm to the nm, *Vaceletia* sp. exhibits exquisite biological control over the formation of its CaCO_3_ skeleton. The overall structure comprises a series of chambers terraced one on top of the next (Fig 1). The skeleton is aragonitic CaCO_3_, with some features of the process of skeleton formation previously described [25, 32, 33]. Briefly, an organic framework is first constructed which is thought to be successively substituted by crystalline aragonite. The organic framework consists of proteins and polysaccharides rich in galactose, glucose and fucose, the latter suggesting that bacterial EPS (exopolymeric substances) may be involved in the biocalcification process. This is likely given that the bacterial biomass of an individual *Vaceletia crypta* can be as high as 50% [25]. Related to this observation we have recently demonstrated that another coralline sponge directly employs its’ bacterial community in a biomineralization role [34, 35]. Furthermore Uriz et al [36] suggest that microbial endosymbionts are directly responsible for the precipitation of the calcium carbonate skeleton in the sponge genus *Hemimycale*. Despite this previous work, little is known about the molecular basis of sponge biomineralization in general. The lack of information from this phylogenetically informative group motivated us to address this problem using *Vaceletia* sp. as a model and transcriptomic and mass spectrometry-based proteomics as tools to address the problem. This approach has allowed us to generate a dataset representing what is likely to be the majority of the *Vaceletia* sp. skeleton forming proteome. To our knowledge this is the first such proteome reported for a sponge.

## Materials and Methods

### Sample collection

Specimens of *Vaceletia* sp. were collected by SCUBA diving during the Deep Down Under Expedition (www.deepdownunder.de) at Osprey- and Bougainville Reefs (Coral Sea, Australia) in depths ranging from 5 to 24 m. Samples for RNA extractions were preserved in RNAlater and stored at -20°C. Samples for protein extractions were freeze-dried and stored at -20°C. Before processing, all samples were carefully inspected under a microscope for contaminating organisms which were carefully removed. Samples for protein extraction were then separated into head and stalk material. Samples were collected during the Deep Downunder excursion under permit number AU-COM2009061.

### Generation of a *Vaceletia* sp. transcriptome

Total RNA derived from the head of one individual was extracted using the miRNeasy Kit (Qiagen) according to the manufacturer’s instructions. Single-end and paired-end Illumina sequencing was conducted using the MiSeq and HiSeq 2000 platforms respectively. A *de novo* transcriptome assembly was performed using Trinity [37], and this dataset was used to conduct all proteomic surveys. All contigs that yielded matches to the MS/MS data can be found in S1 File.

### Matrix preparation

Pools of calcified *Vaceletia* sp. head and stalk pieces (approximately 2 g/pool for head pieces and 4 g/pool for stalk pieces) were treated with sodium hypochlorite (14% active Cl_2_; GPR Rectapur, VWR Chemicals, Germany; 10 ml/g) for 2 h at room temperature with a 5 min ultrasound treatment and changes of hypochlorite solution every 30 min. These pieces were then washed thoroughly with de-ionized water and air-dried. Next, the pieces were placed into a double-layered plastic bag and crushed into smaller pieces in a wrench to liberate the internal structures of the skeleton. The fragments were treated once again with sodium hypochlorite as described above. This treatment was performed in disposable 50 ml centrifuge tubes and skeleton fragments were collected by centrifugation for 5 min at 3000 x g between changes of hypochlorite solution and between washing (3 x) with de-ionized water. The dried skeleton pieces were then demineralization in 50% acetic acid (20 ml/g of sponge skeleton) over night at 4–6°C and the resulting suspension was dialyzed (Spectra/Por 6 dialysis membrane, molecular weight cut-off 2000; Spectrum Europe, Breda, The Netherlands) successively against 3 x 1l of 10% acetic acid and 3 x 1l of 5% acetic acid at 4–6°C. The suspension was then lyophilized. The resulting organic matrices were analyzed by SDS-PAGE using precast 4–12% Novex Bis-Tris gels in MES buffer using reagents and protocols supplied by the manufacturer (Invitrogen, Carlsbad, California), except that 1% β-mercaptoethanol was used as a reducing agent in the sample buffer. Samples were suspended in sample buffer (200 μg / 30 μL), heated to 70°C for 10 min and centrifuged for 5 min at 15,700 x g in a 5415D Eppendorf centrifuge to remove sample buffer-insoluble material. The molecular weight marker was Novex Sharp Pre-stained (Invitrogen). Gels were either stained with the Coomassie using the Colloidal Blue staining kit or silver stained with SilverXpress (both Invitrogen).

### Peptide preparation

Reduction, carbamidomethylation and enzymatic cleavage of matrix proteins were performed using a modification of the FASP (Filter-aided sample preparation) method [38] as outlined below. Aliquots of 200–300 μg of matrix were suspended in 300 μL of 0.1 M Tris, pH 8, containing 6 M guanidine hydrochloride and 0.01 M dithiothreitol (DTT). This mixture was heated to 56°C for 60 min, cooled to room temperature, and centrifuged at 14,000 x g in an Eppendorf bench-top centrifuge 5415D for 15 min. The supernatant was loaded into an Amicon Ultra 0.5 ml 30 K filter device (Millipore; Tullagreen, Ireland). DTT was removed by centrifugation at 14,000 x g for 15 min and washing with 2 x 1 vol of the same buffer. Carbamidomethylation was done in the device using 0.1 M Tris buffer, pH 8, containing 6 M-guanidine hydrochloride and 0.05 mM iodoacetamide and incubation for 45 min in the dark. Carbamidomethylated proteins were washed with 0.05 M ammonium hydrogen carbonate buffer, pH 8, containing 2 M urea, and centrifugation as before. Trypsin (2 μg, Sequencing grade, modified; Promega, Madison, USA) was added in 40 μL of 0.05 M ammonium hydrogen carbonate buffer containing 2 M urea and the devices were incubated at 37°C for 16 h. Peptides were collected by centrifugation and the filters were washed twice with 40 μL of 0.05 M ammonium hydrogen carbonate buffer and twice with 1% trifluoroacetic acid in 5% acetonitrile. The acidic peptide solution (pH 1–2) was applied to C18 Stage Tips [39] and the eluted peptides were vacuum-dried in an Eppendorf concentrator.

### LC-MS and data evaluation

Peptide mixtures were analyzed by on-line nanoflow liquid chromatography using the EASY-nLC 1000 system (Proxeon Biosystems, Odense, Denmark, now part of Thermo Fisher Scientific) with 50 cm capillary columns of an internal diameter of 75 μM filled with 1.8 μM Reprosil-Pur C18-AQ resin (Dr. Maisch GmbH, Ammerbuch-Entringen, Germany). Peptides were eluted with a linear gradient from 5–30% buffer B (80% acetonitrile in 0.1% formic acid) in 90 min, 30–60% B in 5 min and 60–95% B in 5 min at a flow rate of 250 nL/min and a temperature of 50°C. The eluate was electro-sprayed into an Orbitrap Q Exactive (Thermo Fisher Scientific, Bremen, Germany) using a Proxeon nanoelectrospray ion source. The instrument was operated in a HCD top 10 mode essentially as described [40]. The resolution was 70,000 for full scans and 17,500 for fragments (both specified at m/z 400). Ion target values were 1e6 and 5e4ms, respectively. Dynamic exclusion time was 20 sec or 10 sec. MS runs were monitored using the SprayQC quality monitoring system [41]. Raw files were processed using the Andromeda search engine-based version 1.5.0.8 of MaxQuant (http://www.maxquant.org/) with enabled second peptide, iBAQ, and match between runs (match time window 0.5 min; alignment time window 20 min) options [42, 43]. The sequence database was combined with the reversed sequences for FDR calculation and sequences of common contaminants, such as human keratins and mammalian cytoskeletal proteins. Carbamidomethylation was set as fixed modification. Variable modifications were oxidation (M), N-acetyl (protein), pyro-Glu/Gln (N-term), phospho (STY), and hydroxyproline. The initial mass tolerance for full scans was 7 ppm and 20 ppm for MS/MS. Two missed cleavages were allowed and the minimal length required for a peptide was seven amino acids. Maximal FDR for peptide spectral match, proteins and site was set to 0.01. The minimal score for peptides was 60 and the minimal delta score for modified peptides was 17. Identifications with only one or two sequence-unique peptides identified at least 10 and three times, respectively, were routinely validated using the MaxQuant Expert System software [44] considering the assignment of major peaks, occurrence of uninterrupted y- or b-ion series of at least four consecutive amino acids, preferred cleavages N-terminal to proline bonds, the possible presence of a2/b2 ion pairs and immonium ions, and mass accuracy. The iBAQ (intensity-based absolute quantification) [45] option of MaxQuant was used to calculate, based on the sum of peak intensities, the approximate share of each protein in the total proteome, including identifications, which were not accepted after manual validation. This enabled us to discern between minor and major proteins.

Sequence similarity searches were performed with FASTA (http://www.ebi.ac.uk/Tools/sss/fasta/) [46] against current releases of the Uniprot Knowledgebase (UniProtKB). Other bioinformatics tools used were Clustal Omega for sequence alignments (http://www.ebi.ac.uk/Tools/msa/clustalo/) [47], InterPro (http://www.ebi.ac.uk/interpro) [48] for domain predictions, SignalP 4.1 (http://www.cbs.dtu.dk/services/SignalP/) [49] for signal sequence prediction, and TMHMM 2.0 (http://www.cbs.dtu.dk/services/TMHMM-2.0) [50] for transmembrane sequence prediction. Amino acid composition and theoretical pI were determined using the ProtParam tool provided by the Expasy server (http://web.expasy.org/protparam/) [51]. Intrinsically disordered protein structure was predicted using IUPred (http://iupred.enzim.hu/) [52]. Subcellular location predictions were based on sequence similarities to known proteins, domain predictions, signal sequence predictions, and transmembrane segment predictions.

### Spherulin Sequence alignment and Phylogenetic analysis

An alignment of Spherulin homologs was constructed using the dataset described in Jackson et al [35]. The *Vaceletia* sp. sequence was included in this collection of sequences and aligned as previously described. Phylogenetic analyses were conducted using MrBayes v. 3.2.3 and the following parameters: lset nst = 6 rates = invgamma; prset applyto = all; mcmcp nruns = 4, ngen = 1000000, relburnin = yes, burninfrac = 0.25, printfreq = 1000, samplefreq = 100, nchains = 4, temp = 0.2, savebrlens = yes. The first 25% of these trees were discarded as burn in.

### Histology

Fixed *Vaceletia sp*. material was decalcified, dehydrated, embedded in paraffin and sectioned at 2–5 μM. Sections were deparaffinized and stained using alcian-blue fast red dye [53].

### Comparison of *Vaceletia* sp. biomineral proteins to other sponge transcriptomes

TBLASTX based comparison of selected *Vaceletia* sp. proteins were made against eight previously published sponge transcriptomes [54]. Those *Vaceletia* sp. sequences that shared similarity with any contigs within the eight 'Riesgo' transcriptomes were then searched against the NCBI-UniProt/Swissprot database using BLASTX. All BLAST searches were performed using an e-value cut-off of 1e^-5^. HMMER v3.1b2 (www.hmmer.org) and CD-Search [55] were used to screen for protein domains against the Pfam 28.0 Protein Family database [56] and the CDD database v3.14 [57], respectively.

## Results and Discussion

**Fig 2:**
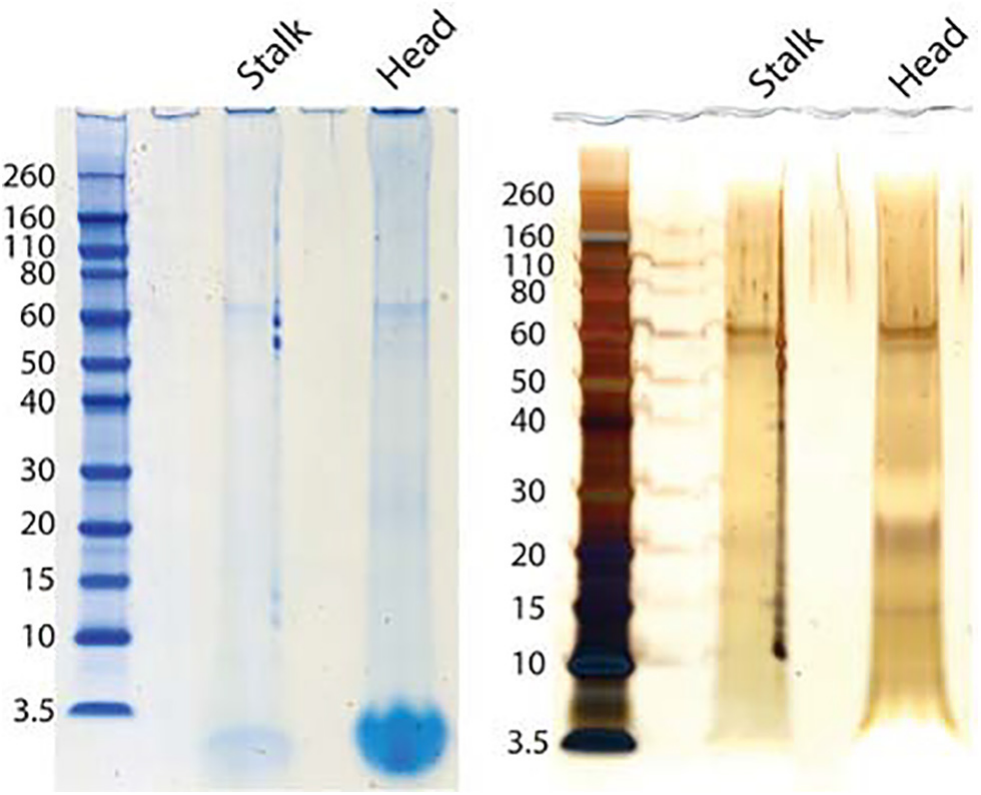
PAGE separation of stalk (S) and Head (H) skeleton matrix. The same gel was first stained with Coomassie Brilliant Blue (CBB, left) and then with silver (right). The molecular weight of marker proteins is given in kDa.

**Fig 3:**
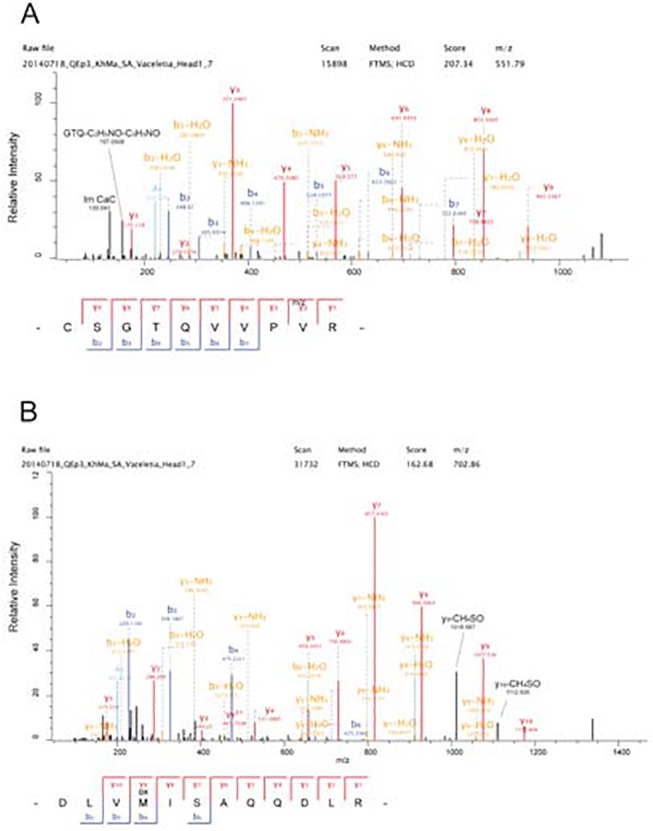
Selected spectra of single sequence-unique peptide identifications. Y-ions are shown in red, b-ions in blue, a-ions in light blue, b- and y ions showing loss of water or ammonia are shown in orange, ions annotated with the help of the MaxQuant Expert System are shown in black. (A) peptide of entry c102844_g1_i1_3. Two fragments annotated with the help of the Expert system are the immonium ion of carbamidomethylated cysteine (Im CaC) and an internal fragment at m/z 157.0508 derived from the tripeptide GTQ. (B) peptide of entry C41414_g3_i1_2. Ions y10 and y9 show the loss of CH_4_SO typical for oxidized methionine residues (Met-sulfoxides).

The yields of organic matrix/g of skeleton were 2.4 mg for head and 2.2 mg for stalk. This value was in good agreement with matrix yields of invertebrate biomineral matrices reported previously [4, 6, 7, 9, 58]. However, PAGE analysis of the matrix proteins yielded a different outcome (Fig 2). Coomassie Brilliant Blue staining showed only very few faint bands that became more prominent with silver staining. This indicated that most of the matrix was either not soluble in PAGE sample buffer or that most of the matrix was not protein. For protein cleavage and peptide isolation under denaturing conditions we used FASP [38], a gel-independent method. The number of identified proteins was low. The head matrix yielded 203 proteins (S2 File: ProteinGroups_HEAD) and the stalk matrix yielded 105 proteins (S3 File: ProteinGroups_STALK), with 19 identifications unique to stalk matrix in this initial list. In agreement with the relatively low number of proteins we identified very few sequence-unique peptides. In head matrix these were 610 (S4 File: Peptides_HEAD) and in stalk we obtained only 215 (S5 File: Peptides_STALK). Furthermore, 43% of the head matrix proteins and 50% of those of stalk matrix were identifications with only one sequence-unique peptide. Such identifications are not commonly accepted in mass spectrometry-based proteomics, at least with samples containing predominantly or exclusively protein. However, many of these peptides were identified many times. Thus, for instance, entry C53634_gi_i1_1, encoding an uncharacterized very acidic protein, was identified with a single sequence-unique peptide that was identified 213 times altogether. Inspection of the sequence contained in this entry indicated that the identified peptide was most probably the only one that could be detected at all. Therefore this identification clearly was a valid one. In other cases the reasons for identification of only one peptide were less obvious and could have included errors in the database, unanticipated modifications, or the scarcity of protein in these samples. Therefore we decided to provisionally accept identifications with one sequence-unique peptide if this was identified more than 10 times and after manual validation of the spectra with the help of the Expert System that is part of the MaxQuant software package [44]. Fig 3 shows some typical annotated spectra of this kind. After elimination of identifications not conforming to these criteria, and combining identifications apparently belonging to the same protein, we obtained a list of 122 accepted protein identifications (S1 Table: *Vaceletia* sp. skeleton matrix proteins). Identifications that were not accepted are provided in S2–S5 Files.

**Fig 4:**
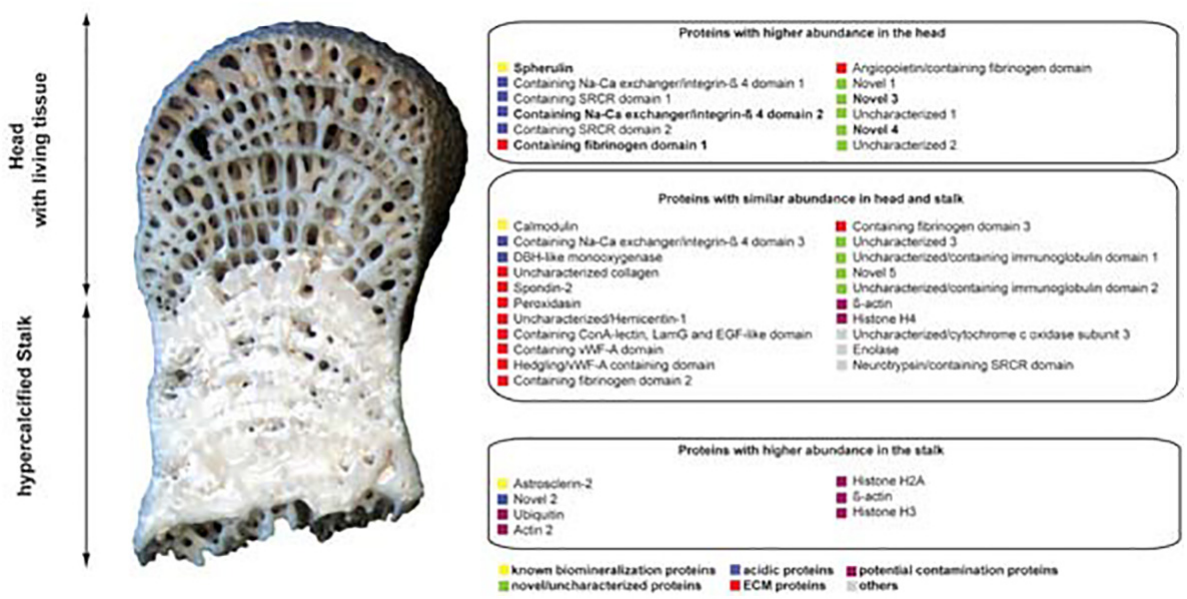
Schematic representation of *Vaceletia sp*. head and stalk region with iBAQ estimates of protein abundances. 12 proteins are enriched in the head, while 7 proteins are enriched in the stalk and 21 proteins have an equal abundance in head and stalk. Protein abundances were considered as different when iBAQ estimations between head and stalk were ≥ 0.5. Proteins in bold were only detected in the head proteome.

All proteins identified in the stalk were present in the head, but some proteins identified in the head were unique to that location. Based on iBAQ values that yield the percentages of proteins normalized to the sum of iBAQ intensities of all identified proteins in a sample, 40 of the 121 identified head proteins constitute more than 90% of the total identified head proteome and 35 of the 72 identified stalk proteins (all of which are present in the head proteome) constitute more than 87% of the stalk total identified proteome (Table 1). We will only consider these 40 "major" proteins further as they are likely to represent the key components of *Vaceletia*'s biomineral proteome, however all 122 isotigs (consisting of 181 contigs) are provided in S1 File. In general the majority of these proteins share similarity with proteins in UniProt and/or contain recognizable protein domains (Table 1); eleven of the 40 most abundant proteins did not return hits against UniProt. Of these 40 major proteins approximately 50% apparently differ in their abundance within the head and stalk regions (19 out of 40; Fig 4).

**Table 1:** The major proteins of the *Vaceletia* sp. head and stalk proteome: 40 proteins (with an iBAQ percentage more than 0.1) constitute more than 90% of the head and more than 87% of the stalk proteome.

Contig	Similarity to	E-value	Protein features	Isoelectric point	% of total in head/stalk (iBAQ)
C7761_g1_i1_1	A6YCJ0 (Sponge)	4.90E-29	Similar to astrosclerin-2; Domain: α-; 11% L; pI 5.6; shares 1 peptide with c94004_g1_i1_2	5.6	31.9 / 51.6
C38723_g1_i1_3	K1WIY3 (Cyanobacteria)	1.80E-08	Similar to Na-Ca exchanger/integrin-β4; domains: Na-Ca-exchanger/integrin_β4; TMH, PM	4	8.8 / 3.2
C99840_g1_i1_1	None	-	(10% G, 11% I, 11% V); TMH	5.2	5.8 / 0.8
C53634_g1_i1_3	None	-	(18% D, 12% E, 13% I, 10% V)	3.5	5.6 / 8.2
C36962_g2_i1_6	W4Y3E1 (Urchin)	1.50E-25	Sp-Srcr85; domain: SRCR, PM	4.5	5.6 / 3.4
C23124_g1_i2_3/g1_i1_3)	None	-	domain: Na-Ca_exchanger/intrgrin_β4	4	5.0 / -
C94004_g1_i1_2	None	-	-	-	4.1 / -
C77644_g1_i1_3	H2Y8G7 (Ascidian)	3.50E-04	domain: fibrinogen_α,β,γ_C_term_glob, subdomain_2; THM; EC	6	2.9 / -
C32287_g1_i1_1	I1G7C7 (Sponge)	3.20E-37	(10% I, 10% S); see also C31462_g1_i1_1	5.7	2.2 / 1.2
C29357_g1_i1_2	A0A022L1D0 (Actinobacteria)	1.10E-06	Uncharacterized collagen (fragment) /α1,6-glucosidase,; domain: triple_helical, EC	9.2	1.9 / 1.8
C22072_g1_i1_3	None	-	15% L; THM	9.2	1.5 / -
C54677_g1_i1_2	Q5QBF8 (Insect)	1.50E-65	Ubiquitin; IC, EC	-	1.3 / 2.7
C3544_g1_i1_1	Q2KT50 (Diatom)	2.40E-32	Actin 2	8.9	1.1 / 1.7
C37591_g1_i3_5	B5X2X5 (Bony Fish)	7.20E-13	Spondin-2; Domain: spondin; EC	8.6	1.1 / 1.2
C1963_g1_i2_2	K1QSR0 *(*Oyster)	6.40E-07	Similar to angiopoietin-4; domain: fibrinogen_α,β,γ_C_term_glob, subdomain_1; EC	5.7	1.1 / 0.4
C20021_g1_i1_2	H2AZL (Frog)	1.10E-40	Histone H2A	9.7	0.9 / 5.2
C36962_g2_i3_6/C80079_g1_i1_2	W4XYX3 (Urchin)	3.00E-20	Sp-Srcr71; domain: SRCR	4.1	0.9 / 0.2
C41075_g1_i4_4/g1_i2_4	V5YU14 (Starfish)	5.90E-160	β-actin; shares 4 peptides with c14026_g1_i1_3 and 1 with c3544_g1_i1 and c21396_g1_i1_4, IC	-	0.8 / 1.9
C32738_g1_i3_3	I1G9M3 (Sponge)	8.10E-08	Uncharacterized; 11% L; TMH	5.5	0.8 / 0.1
C64227_g1_i1_3	None	-	Uncharacterized; domain: fibrinogen _α,β,γ_C_term_glob; 10% L; TMH	8.2	0.7 / 1.1
C102844_g1_i1_3	S9WWY6 (Mammal)	7.40E-12	Similar to neurotrypsin (fragment); domain: SRCR; 13% G, 13% S, 10% V; PM	8.7	0.7 / 0.8
C21396_g1_i1_4	G9I1P2 (Bony Fish)	1.20E-47	Cytoplasmic β-actin (fragment); domain: actin_related (aa1-115); shares peptide with c3544_g1_i1_1 and c41075_g1_i4_4/c41075_g1_i2_4; IC	-	0.6 / 0.6
C80614_g1_i1_3	V6GWB1 (Spirochaetes)	4.60E-18	Similar to peroxidasin; domain: haem_peroxidase	8.8	0.6 / 0.3
C97612_g1_i1_1	K1QE34 (Oyster)	8.50E-11	Similar to DBH-like monooxygenase protein 2-like protein; domain: DOMON	4.2	0.5 / 0.4
C41117_g3_i2_4/g3_i3_5/g3_i1_5/g3_i3_5/g3_i1_5/g3_i4_6	K7LZT4 (Soybean)	2.10E-39	Histone H4; IC	-	0.4 / 0.8
C40964_g7_i1_1/g7_i2_1/g7_i4_2	K1R2Z9 (Oyster)		Uncharacterized/hemicentin-1; domains:metallopeptidase, disintegrin, EGF_3, 6x TSP1; 12% G, 11% S; EC, PM	5.6	0.4 / 0.2
C3160_g1_i1_2/g1_i2_2	H2V0I8 (Bony Fish)	9.80E-08	domains: ConA_lectin/LamG, EGF-like;	6.1	0.4 / 0.2
C35050_g1_i1_1	None	-	Uncharacterized; domain: Na-Ca-exchanger/integrin_β4	4.4	0.3 / 0.5
C40249_g1_i3_3/g1_i2_3/g1_i1_3	A7S664 (Sea Anemone)	2.30E-12	Uncharacterized; domain: VWA	4.9–5.7	0.3 / 0.3
C38115_g2_i1_3/g1_i1_5	I1GHA4 (Sponge)	1.10E-81	Enolase; domains: enolase_N-term, enolase_C-term;TMH; IC, PM	-	0.3 / 0.2
C38911_g1_i3_1	None		Uncharacterized; pI 5.4; domain: PTHR24637; TMH	-	0.3 / 0.2
C100960_g1_i1_4	I1FHH5 (Sponge)	5.20E-05	Similar to Hedgeling/uncharacterized; domain: VWA; PM	-	0.3 / 0.1
C27354_g1_i1_6/C34006_g1_i1_6/g1_i2_6	F6VY96 (Mammal)	1.40E-50	Histone H3 (fragment)	-	0.2 / 0.8
C41693_g1_i7_5/g1_i3_6	I1EQR1 (Sponge)	5.10E-03	Uncharacterized; domain: fibrinogen_ α,β,γ_C_term_glob; EC	5.6	0.2 / 0.1
C41731_g1_i3_5	None	-	(10% I, 12% L, 10% S), TMH	9.2	0.2 / 0.1
C35925_g1_i3_2	None	-	Uncharacterized; domains: IG; 12% S; pI 6.3; TMH	6.3	0.2 / 0.1
C41377_g2_i1_1	None	-	Uncharacterized; domains: IG (58–137), DUF4440 (172–278); shares peptides with c41377_g2_i2_1; TMH	8.5	0.2 / 0.1
C41584_g1_i4_5/g1_i2_5/g1_i5_5	G8HT99 (Stony Coral)	1.70E-05	Uncharacterized/similar to cytochrome c oxidase subunit 3; 10% L, 13% S; shares 1 peptide with C41584_g1_i8_4; TMH	6.6	0.2 / 0.1
C32545_g1_i1_1/g1_i2_1	H6TI88_9METZ (Sponge)	3.70E-33	Spherulin; SSP (aa26/27); EC	4.7	0.1 / -
C103979_g1_i1_6	B5XCM2 (Bony Fish)	8.00E-40	Calmodulin; domain: EFh_pair, shares 1 peptide with C27518_g1_i1_4H; IC	-	0.1 / 0.1

IC = intracellular; EC = extracellular; PM = plasma membrane. TMH = predicted trans-membrane helix.

The most abundant protein in the *Vaceletia* sp. skeletal proteome (Contig 7761) is found at levels more than 10 times that of the next most abundant (Table 1) and shares significant similarity with the Astrosclerins, a family of alpha-carbonic anhydrases (α-CAs) previously identified in another coralline demosponge, *Astrosclera willeyana* [59]. CAs catalyze the reversible reaction of CO_2_ and water to HCO_3_^-^ (which can then react with free Ca^2+^ to form CaCO_3_) and are known to play an important role in invertebrate biomineralization [60, 61]. Astrosclerin is directly involved in the formation of the hypercalcified aragonitic skeleton of *A*. *willeyana* and is also highly expressed in that sponge [59]. The *Vaceletia* sp. α-CA homolog constitutes more than 30% of the head proteome and more than 50% of the stalk proteome, suggesting that this protein is also a key component of *Vaceletia*’s biomineralization toolkit.

Jackson and co-workers also identified another protein involved in biomineralization in *Astrosclera willeyana* that is present in *Vaceletia* sp.’s skeleton. Spherulin is expressed in the same spherulite forming cells as Astrosclerin, and was most likely acquired via a horizontal gene transfer (HGT) event from a prokaryote [35]. *Vaceletia* sp. isotig 32545 (possibly represented by two contigs) shares significant similarity with the Awi-spherulin. Interestingly it is only present in *Vaceletia* sp.’s head proteome in minor quantities (0.1% of total head iBAQ). *Vac*-spherulin only returns hits against bacterial proteins with similarities to sugar transporters. This finding is consistent with the hypothesis that an HGT event delivered this gene into the genome of a common ancestor of *Astrosclera willeyana*, *Vaceletia* sp., *Amphimedon queenslandica*, *Chondrilla nucula*, *Spongilla lacustris* and the hexactinellid *Aphrocallistes vastus* and was subsequently co-opted to a biomineralization role in *A*. *willeyana* and *Vaceletia* sp.. Our phylogenetic analyses support this interpretation with all sponge spherulins clustering together and the bacterial orthologues forming well separated clades (S6 File). We would like to point out here that although *A*. *willeyana* and *Vaceletia* sp. display very different skeletal morphologies, they apparently share at least two important biomineralization proteins (Astrosclerin and Spherulin). These underlying molecular commonalities should be taken into account when considering the broad evolutionary picture of biomineralization and the apparent plasticity of skeletal morphologies. To elucidate this intriguing question further more data from *Astrosclera willeyana* and other calcifying sponges with divergent skeletal morphologies is required.

### Extracellular matrix proteins (ECM)

Some proteins extracted from *Vaceletia* sp.’s skeletal proteome can be identified as ECM proteins or share similarity with previously characterized biomineralization proteins. c40964 shows similarity to hemicentin-1, a cell adhesion protein that recently has been reported as a soluble organic matrix protein (SOMP) from the coral *Acropora millepora* [62], while c80614 shows similarities to peroxidasin which has been suggested to cross-link proteins in the extracellular space [63]. c37591 contains two spondin domains which are known to function as extracellular neuroregulators by guiding axon growth [64]. Recently, spondins have also been found to play a role in processes associated with bone mineralization; F-spondin seems to be involved in the regulation of bone metabolisms resulting in a high bone mass phenotype in F-spondin deficient mice [65]. All spondins involved in bone metabolism contain six thrombospondin-type 1 domains at their C-terminus, which are absent in the detected *Vaceletia* sp. protein. Spondin-2, also called mindin, has been proposed to function in mice as a pattern-recognition ECM molecule involved in opsonization of microbes for phagocytosis, and is therefore essential to the initiation of the innate immune response [66]. *Vac-*c37591 may therefore either be directly involved in the biomineralization process or provide the skeleton with the ability to resist microbial action.

Although many of the proteins we have identified in *Vaceletia* sp.’s skeleton share no overall similarity with proteins in UniProt, they do contain recognizable domains that are associated with extracellular matrix and/or biomineralization proteins. For example ConA_lectin, laminin G and epidermal growth factor-like (EGF) domains have been reported from the mineralizing matrix of scleractinian corals [62]. Two *Vaceletia* sp. proteins (c40249, c100960) contain a von Willebrand factor type A (vWF-A) domain, which are present in biomineral-associated proteins from several organisms. For example Pif, an acidic protein derived from the pearl oyster *Pinctada fucata* possesses a vWF-A domain that has been shown to play an important role in the formation of nacre [67]. In the calcareous sponge *Leucosolenia complicata*, a vWF-A domain is located at the N-terminus of a carbonic anhydrase that plays an important role in sclerite formation [60].

Collagen, a fibrous protein, and Chitin, an amino-polysaccharide, play important roles in a variety of biomineralization systems as they can act as templates upon which mineralization takes place [68]. For example corals use collagens as a mineralization framework (reviewed in [68]), molluscs can employ chitin [69], while keratose sponges use sponge specific collagens to form their organic skeleton [70]. We detected several collagens in *Vaceletia* sp.’s mineral proteome (c27773, c29357, c34834, c36461, c40551, c58706), with one of these (c29357) categorized as a major protein. We also detected a protein with a chitin-binding domain that is only present in the head region albeit at low abundance (c34763). Chitin is known to act as a mineral framework in Verongida sponges [68, 71].

### Acidic proteins

**Fig 5:**
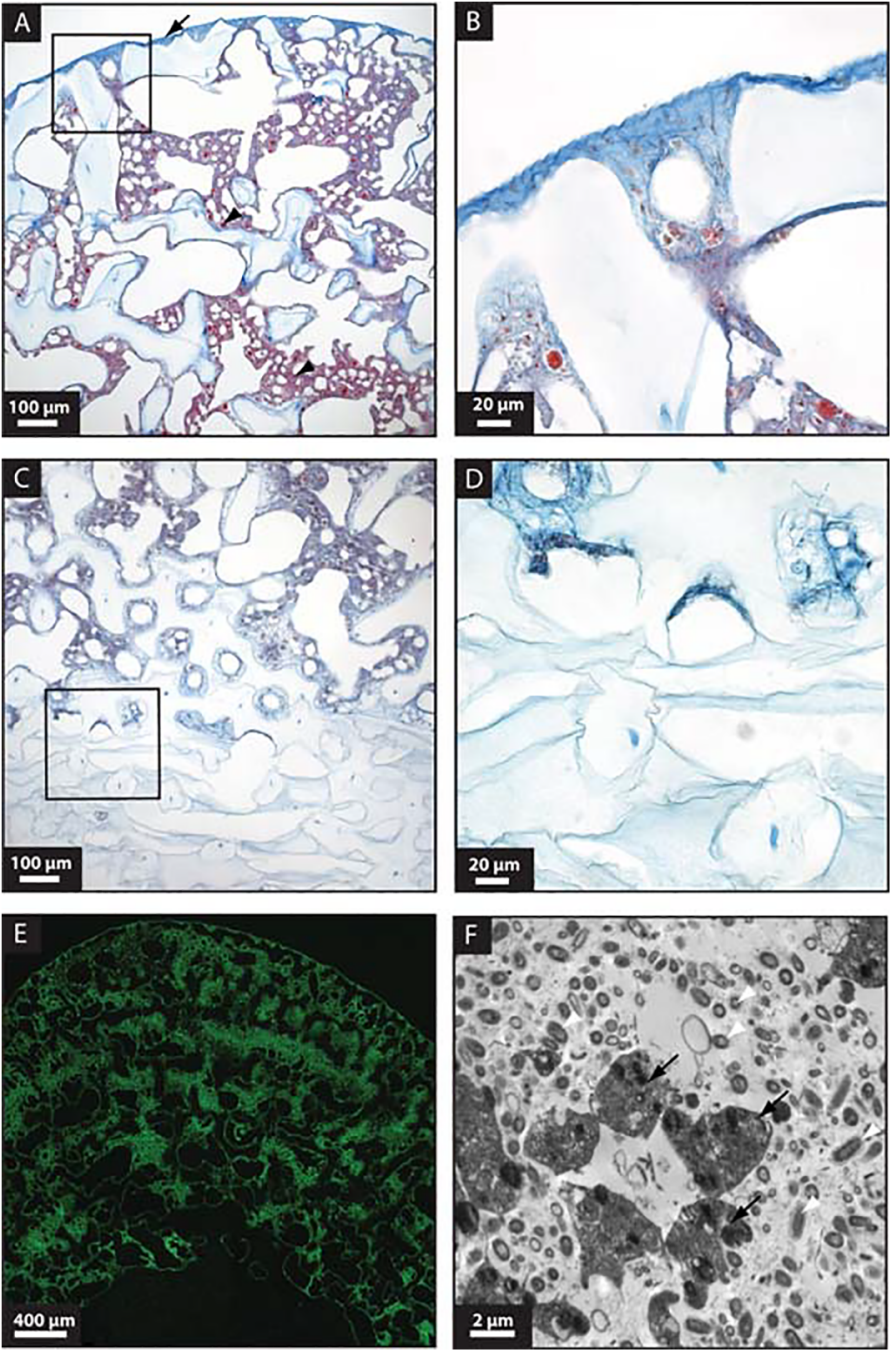
General histological features of decalcified *Vaceletia* sp. (A) Alcian blue stained section of a sagittal sectioned individual illustrating the head region. Blue staining reveals acidic mucopolysacharrides and likely reflects the location of previously calcified pillars (see Fig 1I). Intense red stain reveals sponge larvae (arrowhead) and sponge tissue. (B) Magnification of the boxed section in A illustrates the more intense blue staining in the outermost head region where the acidic substance is produced. (C) Alcian blue staining in the stalk region is less intense than in the head region. (D) Magnification of the boxed section in C shows that the previously calcified stalk region contains no red stained sponge tissue. (E) Sagittally sectioned individual shows the autofluorescent sponge tissue in the head region and a lack of cells in the stalk region. (F) TEM image of sponge mesohyl filled with darkly stained sponge cells (black arrows) and diverse and abundant bacteria (white arrowheads).

Acidic proteins have long been recognized in many organic matrices associated with biomineralization, and it is assumed that they play a major role in this process. Acidic proteins possess a pI < 4.5 [72] and are often rich in negatively charged residues such as aspartic and glutamic acid. They are thought to serve many purposes such as promoting the nucleation of calcium carbonate, determining the growth axes and inhibiting crystal growth [73]. The seven acidic proteins detected in *Vaceletia* sp.’s skeletal proteome account for a large proportion of the total proteome (27% of the total head proteome and 16% of the total stalk proteome). Three of these proteins contain a sodium-calcium-exchanger domain/integrin domain, suggesting a role for binding and delivering calcium to the site of mineralization. Two other acidic proteins contain a scavenger receptor cysteine-rich domain. Proteins containing this domain have been reported from the sea urchin test and spine proteomes [6]. Another acidic protein shows no significant similarity to any UniProt entry but is enriched in glutamic and aspartic acid (18% and 12% respectively). Given their abundance in *Vaceletia* sp.’s skeletal proteome we visualized the spatial distribution of acidic molecules by using an alcian blue + direct-red stain on sections of *Vaceletia* sp.. This staining reveals the insoluble organic framework of the skeleton and an abundance of acidic mucus substances throughout the head and stalk regions with more intense staining in the head region (Fig 5A–5D).

### Uncharacterized proteins

Six proteins extracted from *Vaceletia* sp.’s skeleton contain no recognizable domains and show no similarity to UniProt entries or show similarity to uncharacterized proteins. In some cases it was possible to predict a trans-membrane (TM) domain in these novel proteins. Proteins with TM domains have been found in other datasets of skeletal organic matrix proteins (see for instance [58, 62, 74]). It has been proposed that membrane bound and trans-membrane proteins may contribute directly to the biomineralization process via their extracellular domains. However without functional assays (either *in vitro* or *in vivo*) the roles that these proteins play in biomineralization remain unknown. This is the situation for many proteomic studies of invertebrate biominerals and highlights the growing need for the development of such assays.

### Potential contaminants

Intracellular proteins such as actin, myosin and tubulin are often considered to be contaminations in biomineral protein datasets [75]. According to current working models of biomineralization such skeletal proteomes should only include proteins that are intimately associated with (or occluded within) the mineral phase. Intracellular components such as those listed above have been suggested to derive from cellular debris that remains following inadequate cleaning of the biomineral (i.e. a technical artifact), or that has been inadvertently occluded within the biomineral during its formation [75]. We detected several proteins that fall within this category, namely actin, ubiquitin and histones. In all cases, the abundance of these proteins is higher in the stalk than in the head. *Vaceletia* sp.’s mode of growth may explain this observation. The living tissue of the head lays down new mineral material in the 'outermost' region of the animal (Fig 1C). As growth ensues, older, more proximal regions of the skeleton, continue to mineralize until they are completely fused into the stalk region (Fig 1M–1P). There is no living tissue in the stalk (Fig 5C–5E), so we assume that there would be some degree of apoptosis or unregulated cell death in the region that borders the head and stalk regions. We suggest that cell death in this region may be the source of the higher abundance of these 'contaminating' proteins. Further investigation using cell death and proliferation markers would help to resolve this issue. Of course the alternative interpretation is that these proteins may be genuine biomineralization components. Indeed it has been previously shown that actin and ubiquitin may be involved in the formation of mineralized body parts [76–78]. However we prefer the former interpretation given the lack of living tissue in the stalk region.

### Microbes apparently play a minor proteomic role in skeleton formation in *Vaceletia* sp.

Many sponges are host to a high diversity and large quantity of microbes and species of *Vaceletia* are no exception [79] (Fig 5F). Uriz et al. recently demonstrated that specific bacteria play a direct role in the biomineralization strategy of demosponges belonging to the genus *Hemimycale* [36]. The skeleton of *Hemimycale* sponges is composed of calcitic spherules that are produced by endosymbiontic 'calcibacteria'. Given the deep evolutionary association between sponges and microbes, it was not surprising that a HGT event was detected within the skeleton of another calcifying demosponge *A*. *willeyana* [35], and that we can identify this same gene product in the skeleton of *Vaceletia* sp.. Unexpectedly, and despite the fact that *Vaceletia* sp. is host to a vast community of various microbes, our proteome data contains no evidence that proteins synthesized by symbiotic microorganisms are directly involved in the process of biomineralization within this sponge. However this does not exclude the possibility that microbes may contribute to *Vaceletia* sp.'s biomineralization strategy via other metabolic pathways. Unfortunately developing this line of research further is challenged by the technical limitations of working with these kinds of animals; they are difficult to maintain (let alone culture) in aquaria over long periods of time (necessary in order to observe meaningful skeletal growth), they are found in remote localities, and few functional molecular tools have been developed for them.

### Comparison of *Vaceletia* sp. biomineral proteins to other sponge transcriptomes

Very little work has been done to investigate the molecular biomineralization strategy of sponges in general, and the absence of any other sponge biomineral proteomes prevents the investigation of any potential broad commonalities employed by sponges to build their skeletons. Riesgo et al. [54] recently reported the characterization of eight sponge transcriptomes and while these datasets were not focused on the identification of biomineralization proteins we conducted a survey of these resources using 20 *Vaceletia* sp. biomineralization proteins that were selected on the basis of their high abundance (≥ 1% of the total head proteome) or their potential role in the biomineralization process. Unlike *Vaceletia*, six of the eight sponges employ silica as a primary biomineral (*Aphrocallistes vastus*, *Chondrilla nucula*, *Petrosia ficiformis*, *Spongilla lacustris*, *Pseudospongosorites suberitoides*, *Corticum candelabrum*), while *Ircinia fasciculata* possesses a solely fibrous skeleton and *Sycon coacatum* is the only species to use calcium carbonate to build its skeleton.

Of the 20 *Vaceletia* sp. proteins approximately 50% shared similarity (at an e-value threshold of 1e-5) with one or more proteins derived from the eight sponge transcriptome datasets (S2 Table). A key component of *Vaceletia’s* biomineralization toolkit, a carbonic anhydrase similar to Astrosclerin, is present in seven out of the eight transcriptomes. Besides the role of CA in biomineralization, CA enzymes are also involved in a variety of other metabolic processes such as CO_2_ transport and pH and ion regulation [80, 81]. CA has been identified as a key enzyme employed in the biomineralization strategy of another *Sycon* species *S*. *ciliatum* [60], and it is therefore likely to be involved in the mineralization process of *S*. *coacatum*.

Interestingly, we were able to detect the previously described horizontally transferred gene *Spherulin* [35] in the hexactinellid sponge *A*. *vastus* and in two demosponges, *C*. *nucula* and *S*. *lacustris* from the dataset of Riesgo [54], but could not detect it in the calcifying *S*. *coacatum*. It is tempting to speculate that besides playing a role in sponge calcification [35] Spherulin may also be involved in biosilification. However, the function of Spherulin remains unknown and without further data this must remain speculation. The absence of Spherulin in *S*. *coacatum*, *A*. *vastus*, *P*. *ficiformis*, *P*. *suberitoides*, *C*. *candelabrum* and *I*. *fasciculata* may either indicate the loss of this gene in these species or a lack of expression in the Riesgo [54] transcriptome datasets.

The majority of the 20 *Vaceletia* sp. biomineralizing proteins used in this comparison share similarity to domains present in contigs represented in all eight of the Riesgo transcriptomes (S2 Table). However on the basis of these sequence similarity results it is problematic to infer any genuine homology to the *Vaceletia* sp. biomineralizing proteins we report here; while proteins may share recognizable domains that confer a similar function to the entire protein, this does not necessarily imply that those proteins share an evolutionary history and so we interpret the results of these comparisons with caution.

## Conclusion

The proteome that we report here for *Vaceletia* sp. is the first comprehensive biomineralization dataset from a sponge. As reported for other biomineralization proteomes it contains proteins known to play roles in biomineralization, and novel proteins that display no similarity to known proteins. The presence of deeply conserved biomineralization proteins such as α-CA illustrates that the LCAM did indeed contribute some biocalcification tools to its descendants, and that therefore there is likely to be considerable conservation in the molecular details of skeleton formation across the Metazoa despite divergent skeletal morphologies. Our data suggests that different mineralization processes are taking place in the head and stalk regions, and that bacteria apparently contribute minimal proteinaceous resources directly to the construction of *Vaceletia* sp.'s skeleton. Skeletogenic proteome surveys are important resources that serve to both expand our knowledge of the protein repertoires animals use to biomineralize, and how this ability evolved. However, the lack of assays available to study the functional roles that these proteins play remains a major challenge to the field of biomineralogy.

## Supporting Information

S1 File Contigs coding for proteins identified in either the head or stalk regions of the *Vaceletia* skeleton. (TXT)

S2 File Protein Groups for *Vaceletia* head region. (XLS)

S3 File Protein Groups for *Vaceletia* stalk region. (XLS)

S4 File Peptides for *Vaceletia* head region. (XLS)

S5 File Peptides for *Vaceletia* stalk region. (XLS)

S6 File Bayesian phylogenetic analysis of eukaryotic and prokaryotic spherulin sequences. Posterior probabilities are indicated for each node.
(PDF)

S1 Table Complete list of *Vaceletia sp*. accepted identifications for skeleton matrix proteins. (DOCX)

S2 Table Comparison of *Vaceletia* sp. biomineral related proteins against eight sponge transcriptome datasets. (PDF)
