# Supplementary figures and images for: The Skeleton Forming Proteome of an Early Branching Metazoan: A Molecular Survey of the Biomineralization Components Employed by the Coralline Sponge Vaceletia Sp
Source: PLoS One. 2015 Nov 4;10(11):e0140100. doi: 10.1371/journal.pone.0140100 (PMC4633127; doi:10.1371/journal.pone.0140100)

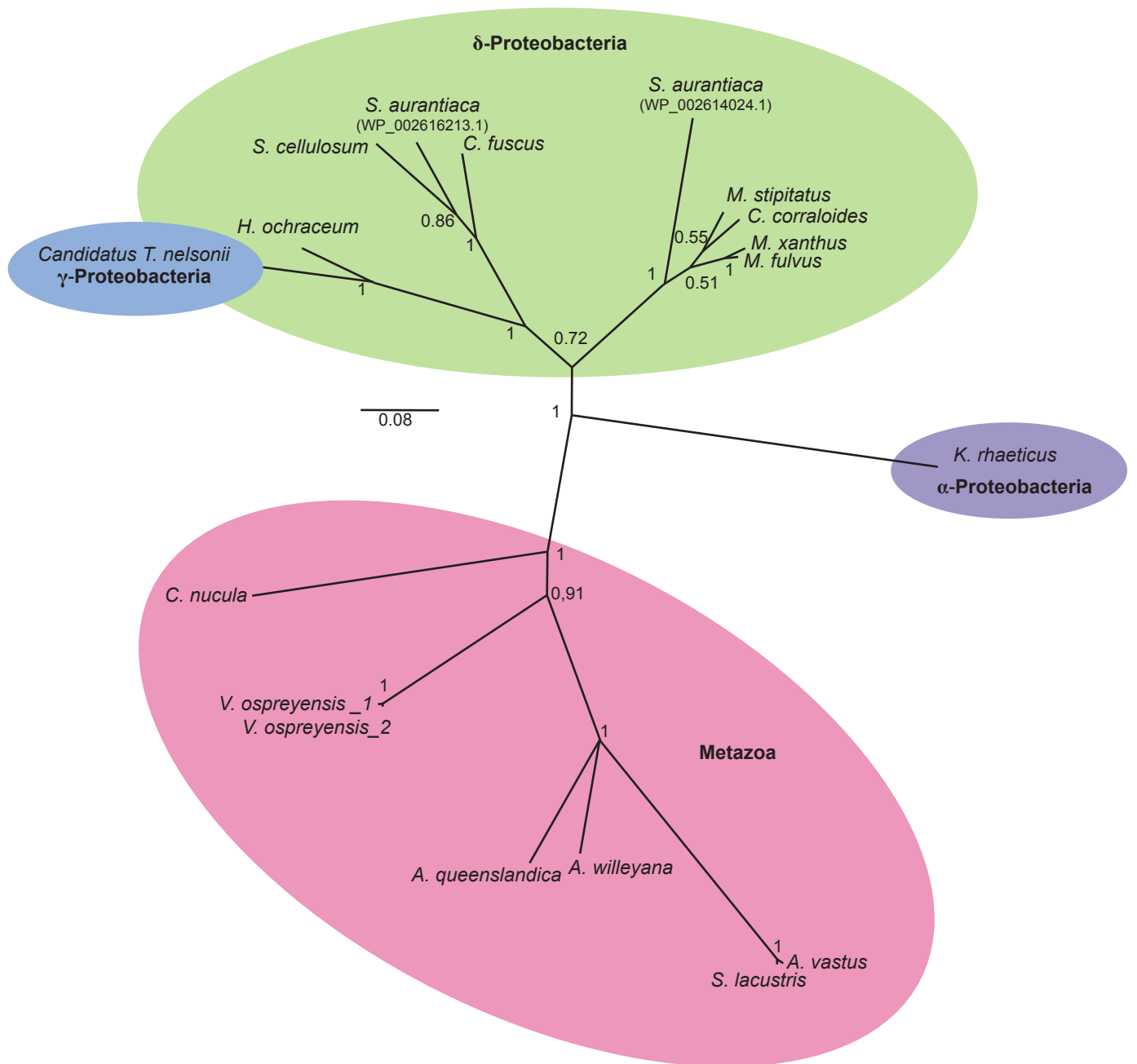

Supplement: S6 File — Posterior probabilities are indicated for each node. (PDF) [file pone.0140100.s006.pdf]
