# Supplementary material for: The Skeleton Forming Proteome of an Early Branching Metazoan: A Molecular Survey of the Biomineralization Components Employed by the Coralline Sponge Vaceletia Sp
Source: PLoS One. 2015 Nov 4;10(11):e0140100. doi: 10.1371/journal.pone.0140100 (PMC4633127; doi:10.1371/journal.pone.0140100)
Supplement: S1 Table — (DOCX) [file pone.0140100.s007.docx]

**S1 Table: *Vaceletia ospreyensis* skeleton matrix proteins**

| **Contig** | **Protein/sequence features** | **Most similar**  (e-value <1.0e-4) | **% iden-tity** | **e-value** | **Head (H)**  **or Stalk (S)** | **Unique and razor pep-tides** | **Total pep-tides** | **% of total (iBAQ)^1^** | **Known sub-cellular**  **loca-tion**^2^ |
| --- | --- | --- | --- | --- | --- | --- | --- | --- | --- |
|  |  |  |  |  |  |  |  |  |  |
| C100552_g1_i1_1 | Uncharacterized; domain: AMMOP; 11% S; pI 6.1 | I1FM43_AMPQE  (aa721-796)  I1F7H4_AMPQE  (aa702-777) | 38.5  37.2 | 2.3e-5  2.3e-5 | S  H | -  2 | -  4 | -  <0.1 | EC |
| C100960_g1_i1_4 | Similar to Hedgeling/uncharacterized; domain: VWA; 11% A, 14% R, 10% G, 11% T; pI 11.1 | A8YRX2_AMPQE  (aa222-292)  I1FHH5_AMPQE  (aa222-292) | 43.7  43.7 | 5.2e-5  5.2e-5 | S  H | 1  2 | 5  27 | 0.1  0.3 | PM |
| C102444_g1_i1_2 | Proteasome subunit alpha type | Q5XGP0_XENLA  (aa42-137) | 100.0 | 3.5e-41 | S  H | -  2 | -  5 | -  <0.1 | IC |
| C102844_g1_i1_3  (aa1-55) | Similar to neurotrypsin; domain: SRCR; 13% G, 13% S, 10% V; pI 8.7 | S9WWY6_9CETA  (aa153-207) | 58.2 | 7.4e-12 | S  H | 1  1 | 18  24 | 0.8  0.7 | PM |
| C103979_g1_i1_6  (aa8-123) | GSONMT00012740001/similar to  calmodulin; domain: EFh; shares 1 peptide with C27518_g1_i1_4 | A0A060WVU0_  ONCMY  (aa30-148) | 98.3 | 5.0e-40 | S  H | 4  4 | 7  15 | 0.1  0.1 | IC |
| C13466_g1_i1_5  (aa19-200) | Marine sediment metagenome DNA, contig S12H4_L01837 (fragment); domain: serpin (aa2-200); 11% L, 10% S; pI 6.4 | X1R7Z1_9ZZZZ  (aa79-262) | 34.0 | 9.0e-19 | S  H | -  3 | -  20 | -  <0.1 | ? |
| C14026_g1_i1_3 | β-actin; shares 4 peptides with c41075_g1_i4_4/c41075_g1_i2_4 | F1DFL6_ANDDA  (aa75-282) | 99.5 | 1.4e-87 | S  H | 2  2 | 8  9 | 0.3  0.1 | IC |
| C14591_g1_i1_3  C35582_g1_i1_5  (aa2-604) | Uncharacterized/similar to α-actinin; domain: calponin (CH); pI 5.5  Uncharacterized;domains: spectrin-repeat, EFh; 10% E, 11% L; pI 4.8 | I1FUQ5_AMPQE  (aa122-278)  (aa279-882) | 86.0  60.6 | 1.1e-57  1.7e-119 | S  H  S  H | -  1  -  4 | -  2  -  12 | -  <0.1  -  <0.1 | IC |
| C18914_g1_i1_1  (aa6-139) | Histone H2B | K7E461_MONDO  (aa4-131) | 80.6 | 6.7e-38 | S  H | 2  4 | 54  22 | 1.2  0.1 | IC |
| C1963_g1_i2_2  (aa14-95) | Similar to angiopoietin-4; 10% G; pI 5.7; domain: fibrinogen_α,β,γ_C_term_glob, subdomain_1 | K1QSR0_CRAGI  (aa239-316) | 41.0 | 6.4e-7 | S  H | 1  1 | 22  51 | 0.4  1.1 | EC |
| C20021_g1_i1_2  (aa1-108) | Histone H2A | H2AZL_XENTR  (aa21-128) | 99.1 | 1.1e-40 | S  H | 3  3 | 15  21 | 5.2  0.9 | IC |
| C21396_g1_i1_4  (aa1-111) | β-actin; shares 1 peptide with c3544_g1_i1_1 and c41075_g1_i4_4/c41075_g1_i2_4 | G9I1P2_SPAAU  (aa22-132)  F1DFL6_ANDDA  (aa264-374) | 100.0  100.0 | 1.2e-47  2.6e-47 | S  H | 3  3 | 33  56 | 0.6  0.6 | IC |
| C22072_g1_i1_3 | Uncharacterized; TMHs; 16.5% L; pI 9.2; domain: IG 8aa234-290; TMH: aa4-26, 94-113, 123-145, 221-243 |  |  |  | S  H | -  1 | -  11 | -  1.5 | ? |
| C23124_g1_i2_3  C23124_g1_i1_3 | Uncharacterized; domain: Na-Ca_exchanger/integrin_β4 (aa11-52); 11% I; pI 4.0 |  |  |  | S  H | -  1 | -  12 | -  5.0 | PM |
| C26034_g1_i1_1  (aa1-83) | Uncharacterized; 10% G, 10% T; pI 7.6 | F2UHE7_SALR5  (aa265-347) | 39.5 | 5.0e-9 | S  H | 1  2 | 6  10 | <0.1  <0.1 | ? |
| C27354_g1_i1_6  C34006_g1_i1_6  C34006_g1_i2_6 | Histone H3 | F6VY96_ORNAN | 98.6 | 1.4e-50 | S  H | 2  2 | 9  6 | 0.8  0.2 | IC |
| C27518_g1_i1_4  (aa1-169) | Similar to calmodulin; domain: EFh_pair; shares 1 peptide with C103979_g1_i1_6 | G4VT44_SCHMA  (aa15-183)  A0A060WVU0_  ONCMY  (aa34-181) | 87.0  93.9 | 1.6e-47  8.4e-46 | S  H | 1  1 | 9  12 | <0.1  <0.1 | IC |
| C27773_g1_i1_3  (aa1-440) | Uncharacterized collagen; domains. triple-helical (aa3-229), C_term (aa253-443), TMH (aa450-472);19% G, 12% P; pI 6.9 | H2VE20_TAKRU  (aa1244-1724) | 34.7 | 7.2e-26 | S  H | -  4 | -  11 | -  <0.1 | EC |
| C28179_g1_i1_4  (aa10-273) | Uncharacterized; domain: fibrinogen_α,β,γ_C_term_glob, subdomain_1 8aa7-64); pI 6.7 | I1FRX7_AMPQE  (aa196-461) | 35.3 | 6.3e-36 | S  H | -  2 | -  10 | -  <0.01 | EC |
| C29357_g1_i1_2  (aa14-149) | α1,6-glucosidase7uncharacterized collagen, 13% G, 10% K; pI 9.2; domain: triple_helical (aa92-147) | A0A022L1D0_ 9MICO  (aa1898-2035) | 36.0 | 1.1e-6 | S  H | 3  3 | 32  43 | 1.8  1.9 | EC |
| C29680_g1_i2_1  (aa6-121)  C29680_g1_i3_1  C29680_g1_i1_1 | Similar to von Willebrand factor D and EGF domain-containing protein (VWDE); domains: EGF, IG; 10% C, pI 7.5 | I3LR65_PIG  (aa1436-1552) | 45.0 | 5.7e-14 | S  H | 1  3 | 4  11 | 0.1  0.1 | EC |
| C31300_g1_i1_1  C31300_g1_i2_3 | Uncharacterized; 10% I, 11% L; TMH: aa10-32, 310-332, 342-364 |  |  |  | S  H | -  2 | -  3 | -  0.01 | ? |
| C31462_g1_i1_1  (aa11-580) | Uncharacterized; domains:Na-Ca-exchanger/integrin_β4 (aa1-89), VWD (aa115-290); pI 4.7 | I1G7C1_AMPQE  (aa2462-3029) | 31.9 | 1.3e-30 | S  H | 2  6 | 18  25 | 0.1  0.1 | ? |
| C3160_g1_i1_2  (aa37-192)  C3160_g1_i2_2 | Uncharacterized; domains: ConA_lectin/LamG; 10% N, 11% L; pI 6.1 | H2V0I8_TAKRU  (aa37-193) | 29.6 | 9.8e-8 | S  H | 1  5 | 6  30 | 0.2  0.4 | ? |
| C31750_g1_i1_4 | Uncharacterized; domains: VWA; 10% L; pI 4.2 | A0A0B7AC05_ 9EUPU | 26.8 | 3.1e-5 | S  H | -  2 | -  8 | -  0.1 | ? |
| C31990_g1_i4_4  C31990_g1_i1_4  C31990_g1_i3_4 | Uncharacterized; 11% L; pI 5.4, TMH: aa56-78, 98-120, 287-306, 676-698, 746-768 |  |  |  | S  H | 4  8 | 15  54 | 0.1  0.1 | **?** |
| C32287_g1_i1_1  (aa34-402) | Uncharacterized; 10% I, 10% S; pI 5.7 | I1G7C7_AMPQE  (aa2447-2832) | 38.9 | 3.2e-37 | S  H | 4  7 | 55  133 | 1.2  2.2 | ? |
| C32545_g1_i1_1  (aa10-409)  C32545_g1_i2_1 | Similar to spherulin; SSP (aa26/27); pI 4.7 | H6TI88_9METZ  (aa4-398) | 32.2 | 3.7e-33 | S  H | -  3 | -  22 | -  0.1 | EC |
| C32626_g1_i1_4 | Elongation factor 1-α1 | D3Z3I8_MOUSE  (aa26-184) | 98.7 | 1.2e-36 | S  H | 3  3 | 9  9 | 0.2  0.1 | IC |
| C32738_g1_i3_3  (aa2-184) | Uncharacterized; 11% L; pI 5.5; TMH: aa162-184, 194-216 | I1G9M3_AMPQE  (aa668-868) | 27.5 | 8.1e-8 | S  H | 2  4 | 3  45 | 0.1  0.8 | ? |
| C33066_g1_i1_6  (aa1-160) | Uncharacterized (peptide-containing region); domain: VWA (aa1-50); 10% E; pI 4.8 | I1EGM4_AMPQE  (aa325-476) | 28.5 | 3.7e-7 | S  H | -  2 | -  6 | -  <0.01 | ? |
| C33984_g1_i2_3  (aa65-562) | Uncharacterized; 12% C; pI 6.1 | I1GJ91_AMPQE  (aa404-980) | 25.2 | 1.4e-15 | S  H | 5  10 | 23  60 | 0.1  0.1 | ? |
| C34028_g1_i1_1 | Uncharacterized; pI 9.3 |  |  |  | S  H | -  2 | -  6 | -  <0.1 | ? |
| C34732_g1_i2_1  (aa1-245) | Similar to Wnt; domain: Wnt (aa1-246), TMH (aa260-282,289-311,316-338); pI 5.5 | Q4ADV9_GLARU  (aa105-351) | 41.3 | 1.3e-36 | S  H | -  2 | -  3 | -  <0.1 | EC |
| C34763_g1_i1_1  (aa13-268) | Similar to α2-macroglobulin; domains: chitin_binding (aa72-102), A2M (aa151-240), SSP 8aa30/31); 10% S; pI 6.5 | I3MDI6_SPETR  (aa4-252) | 25.9 | 1.2e-7 | S  H | -  3 | -  16 | -  0.1 | EC |
| C34834_g1_i2_6  (aa1-446)  C34834_g1_i3_6  C34834_g1_i4_4  C34834_g1_i5_6  C34834_g1_i1_4 | Uncharacterized collagen; 27% G, 16% P; pI 5.2 | I1FIU3_AMPQE  (aa666-1127) | 48.0 | 1.2e-40 | S  H | -  3 | -  19 | -  <0.1 | EC |
| C35050_g1_i1_1 | Uncharacterized; domain: Na-Ca-exchanger/integrin_β4 (aa50-83); 15% D, 11% I, 13% V; pI 4.4 |  |  |  | S  H | 2  2 | 11  22 | 0.5  0.3 | ? |
| C3544_g1_i1_1 | Actin(-2); shares 1 peptide with c21396_g1_i1_2 and c41075_g1_i4_4 | Q2KT50_9STRA  (aa87-166) | 96.2 | 2.4e-32 | S  H | 1  2 | 16  20 | 1.7  1.1 | IC |
| C35750_g1_i1_1  (aa1-196) | HSP70; shares 1 peptide with c65284_g1_i1_4 and c94913_g1_i1_1; pI 10.4 | C3S1H7_STIJA  (aa28-223) | 88.3 | 2.4e-68 | S  H | -  3 | -  7 | -  <0.1 | IC |
| C35925_g1_i3_2 | Uncharacterized; domains: IG (aa196-295,383-478); 12% S; pI 6.3; TMH: aa4-26, 38-60 |  |  |  | S  H | 1  1 | 6  20 | <0.1  0.2 | ? |
| C36455_g1_i1_1  (aa1-546) | Uncharacterized; domain: Chondroitin N-acetylgalactosaminyltransferase (aa255-555) | I1GCB6_AMPQE  (aa230-776) | 45.4 | 1.7e-95 | S  H | -  2 | -  4 | -  <0.1 | IC |
| C36461_g1_i1_1 | Uncharacterized collagen; 24% G, 15% P; pI 8.3; domain: triple_helical (aa1-470), fibrillar_collagen_C_term (aa499-681) | M3ZWX1_XIPMA  (aa1066-1748) | 42.7 | 1.5e-49 | S  H | 1  1 | 11  15 | <0.1  <0.1 | EC |
| C36846_g1_i1_5  (aa27-531) | Similar to protein disulfide-isomerase; SSP aa1-45; domains: thoredoxin/disulphide isomerase; 11% E, 10% K; pI 4.8 | B6RB63_HALDI  (aa4-499) | 58.0 | 6.5e-110 | S  H | 2  9 | 10  51 | <0.1  0.1 | IC |
| C36962_g2_i1_6 | Uncharacterized; domain: SRCR (aa12-111); 20% G, 11% S; pI 4.5 | W4Y3E1_STRPU  (aa611-720) | 63.1 | 1.5e-25 | S  H | 1  2 | 27  43 | 3.4  5.6 | ? |
| C36962_g2_i3_6  C80079_g1_i1_2 | Uncharacterized; domain: SRCR (16-75); 20% G, 11% V; pI 4.1 | W4XYX3_STRPU  (aa775-849) | 70.7 | 3.0e-20 | S  H | 1  1 | 3  13 | 0.2  0.9 | ? |
| C37098_g1_i1_6  (aa178-443) | Uncharacterized; domain: fibrinogen_α,β,γ_C_term_glob (aa172-214) | I1GHE9_AMPQE  (aa327-584) | 30.9 | 2.7e-22 | S  H | -  2 | -  9 | -  <0.1 | EC |
| C37261_g1_i1_1  C37261_g1_i3_3  C37261_g1_i4_3 | Similar to cystatin-A; domains: protease inhibitor I24(A); 10% I, 11% V; pI 5.5 | K76FR9_PELSI  (aa4-100) | 54.6 | 1.6e-15 | S  H | -  1 | -  11 | -  0.03 |  |
| C37281_g1_i1_3  (aa2-403) | Similar to lysosomal α-glucosidase; domain: glycoside-hydrolase_31; pI 5.5 | I1G9Z0_AMPQE  (aa493-888) | 49.3 | 2.3e-81 | S  H | -  4 | -  18 | -  <0.1 | IC |
| C37333_g1_i2_1  (aa2-452)  C37333_g1_i1_1 | Similar to Superoxide dismutase [Cu-Zn]; domain: SOD (aa151-317), TMH (aa433-455) | I1GEI9_AMPQE  (aa298-719) | 28.3 | 1.2e-15 | S  H | -  2 | -  11 | -  <0.1 | ? |
| C37405_g1_i1_6  (aa6-552)  C37405_g1_i2_3 | ATP synthase subunit alpha | T1E6G7_CROHD  (aa1-551) | 79.2 | 1.7e-167 | S  H | -  3 | -  7 | -  <0.1 | IC |
| C37486_g1_i1_5  (aa1-361) | Elongation factor 1-α1 | G3HH39_CRIGR  (aa140-503) | 87.1 | 7.9e-129 | S  H | 2  3 | 16  17 | 0.2  0.1 | IC |
| C37591_g1_i3_5  (aa334-501) | Similar to spondin; domains: spondin (aa98-284, 338-523); 10% S; pI 8.6 | B5X2X5_SALSA  (aa31-205) | 31.3 | 7.2e-13 | S  H | 5  8 | 41  76 | 1.2  1.1 | EC |
| C38115_g1_i1_5  C38115_g2_i1_3 | Similar to LOC100633963/enolase; domains: enolase_N-term, enolase_C-term; TMH: aa458-480 | I1GHA4_AMPQE  (aa233-434)  (aa1-238) | 81.7 | 6.6e-72 | S  H  S  H | 1  -  1  1 | 3  -  13  21 | 0.04  -  0.23  0.32 | IC,PM |
| C38191_g1_i1_4  (aa20-300) | Proteasome subunit α- type | T1K2I7_TETUR  (aa1-265) | 65.7 | 3.0e-39 | S  H | 2  2 | 2  5 | <0.1  <0.1 | IC |
| C38606_g1_i2_3  (aa2-188)  C38606_g1_i1_3  C39537_g2_i2_5  (aa1-333) | Uncharacterized/similar to SOD; domain: SOD:copper/zinc_binding (aa3-239); 13% S; pI 5.1  Uncharacterized;domains: SOD, TMH (aa608-630,643-665); pI 5.4 | C3YJ02_BRAFL  (aa15-192)  (aa122-481) | 39.2  27.7 | 2.4e-24  3.2e-13 | S  H  S  H | -  2  -  5 | -  6  -  13 | -  <0.1  -  <0.1 | IC |
| C38723_g1_i1_3  (aa5-312) | Similar to Na-Ca exchanger/integrin-β4; domains: Na-Ca-exchanger/integrin_β4 (aa4-85, 105-205); TMH (aa385-407); 11% V; pI 4.0 | K1WIY3_ARTPT  (aa632-927) | 26.0 | 1.8e-8 | S  H | 2  2 | 48  94 | 3.2  8.8 | PM |
| C38774_g1_i4_5  (aa64-372) | Similar to aqualysin, domains: peptidase_S8 (aa91-361), TMH (aa431-453); 10% L; pI 8.1 | B3PIU6_CELJU  (aa119-418) | 34.9 | 6.4e-26 | S  H | -  2 | -  5 | -  <0.1 | EC |
| C38911_g1_i3_1 | Uncharacterized; pI 5.4; domain: PTHR24637 (aa169-335); TMH: 495-517, 537-559 |  |  |  | S  H | 3  5 | 23  65 | 0.2  0.3 | ? |
| C38968_g1_i2_6  C38968_g1_i3_4 | Uncharacterized; TMH (aa28-50,258-280,290-312); 12% L; pI 5.7 | I1FRM0_AMPQE  (aa62-236) | 27.5 | 1.8e-7 | S  H | -  2 | -  7 | -  <0.1 | ? |
| C38980_g1_i1_3  C38980_g1_i5_3  C38980_g1_i2_3 | Uncharacterized/Glycosyl hydrolase family 25; domains: IG, FN type III, TMH (aa482-504); 11% S, 11% T; pI6.5 | R5VS88_9FIRM | 23.4 | 4.6e-8 | S  H | -  2 | -  3 | -  0.01 |  |
| C39074_g1_i3_2  (aa85-511)  C39074_g1_i3_2  C39074_g1_i3_2 | ATP synthase subunit beta | I2CCV5_9METZ  (aa1-427) | 98.8 | 2.3e-159 | S  H | -  3 | -  3 | -  <0.1 | IC |
| C39107_g1_i1_3  (aa30-755) | Similar to HSP-90A; domains: HSP90_N_term (aa42-251), RSP_S5_2 (315-570); 10% E, 10% K; pI 5.0 | L7MEG0_9ACAR  (aa36-763) | 81.7 | 1.6e-155 | S  H | 3  1 | 9  6 | <0.1  <0.1 | IC,EC |
| C39500_g1_i1_2  C39500_g1_i3_2 | Uncharacterized; pI 8.0; TMH (aa15-37) |  |  |  | S  H | -  4 | -  17 | -  0.1 | ? |
| C39819_g2_i1_4  C39819_g2_i2_5 | Uncharacterized; domains: VWA (aa1-147,194-431), trypsin_inhibitor_like_ cys_rich (aa428-481); pI 4.9 |  |  |  | S  H | 3  8 | 6  48 | <0.1  0.1 | ? |
| C39828_g1_i3_5  (aa12-349)  C39828_g1_i1_6 | Similar to malate dehydrogenase; domains: MD_N-term (aa38-181), MD_C_term (aa181-348); 11% L, 10% S; pI 8.5 | A7T1J0_NEMVE | 69.3 | 5.8e-95 | S  H | 2  3 | 4  9 | <0.1  <0.1 | IC |
| C39985_g1_i1_2  (aa113-413) | Uncharacterized; domain: alkaline_ phosphatase_like/sulfatase; 11% L; pI 6.0; TMH (aa419-441,446-468,478-500) | I1GC70_AMPQE  (aa213-504) | 61.1 | 2.5e-79 | S  H | 1  2 | 13  21 | 0.1  <0.1 | ? |
| C40249_g1_i3_3  (aa2-394)  C40249_g1_i2_3  C40249_g1_i1_3 | Uncharacterized; domain: VWA (aa149-318); pI 4.9-5.7 | A7S664_NEMVE  (aa411-813) | 27.4 | 2.3e-12 | S  H | 2  6 | 24  54 | 0.3  0.3 | ? |
| C40375_g1_i4_5  C40375_g1_i3_4 | Uncharacterized; 12% S; pI 8.6; domains: GPCR (353-421), FN3 (aa569-680) |  |  |  | S  H | 1  4 | 2  20 | <0.1  <0.1 | ? |
| C40380_g1_i3_3  C40380_g1_i1_3  C40380_g1_i5_3  C40380_g1_i4_3 | Uncharacterized, domains: SSP (aa27/28), β/γ_crystallin (aa126-163), TMH (aa366-388,441-463,488-510) |  |  |  | S  H | -  3 | -  5 | -  0.1 | EC |
| C40434_g1_i1_2  (aa9-505)  C40434_g1_i2_2 | Pyruvate kinase | I1GHR0_AMPQE  (aa61-546) | 68.8 | 3.4e-127 | S  H | 2  2 | 4  9 | <0.1  <0.1 | IC |
| C40551_g1_i1_3  (aa9-203) | Uncharacterized collagen; 18% G, 10% P; pI 9.9; domain: triple_helical (aa1-42,132-203) | W5PEV2_SHEEP  (aa1261-1460) | 33.2 | 3.5e-9 | S  H | 2  3 | 8  14 | 0.1  0.1 | EC |
| C40657_g1_i2_4  (aa31-354) | Similar to calumenin/reticulobindin, domains: EFh; TMH (aa28-45); 11% L; pI 5.8 | I1FZZ1_AMPQE  (aa1-323) | 50.0 | 1.8e-65 | S  H | 2  2 | 14  12 | <0.1  <0.1 | IC |
| C40959_g4_i1_2  C40959_g4_i2_2  C40959_g4_i3_2 | Uncharacterized; TMH (aa7-26); 10% Q, 10% E, 12% L; pI 5.6 |  |  |  | S  H | -  2 | -  4 | -  <0.1 |  |
| C40964_g7_i1_1  (aa~350-730)  C40964_g7_i2_1  C40964_g7_i4_2 | Uncharacterized/hemicentin-1; domains:  metallopeptidase (aa1-131), disintegrin (aa114-202, EGF_3 (aa346-382), 6x TSP1 (aa390-735); 12% G, 11% S; pI 5.6 | C3ZHC1_BRAFL  K1R2Z9_CRAGI  (aa~10-400) | 47.5  42.9 | 1.6e-64  2.5e-64 | S  H | 5  7 | 42  78 | 0.2  0.4 | EC,PM |
| C40975_g2_i3_1  (aa1-153) | β-tubulin; shares 2 peptides with C40975_g4_i2_1 | Q26165_PATVU  (aa289-441) | 96.7 | 4.4e-60 | S  H | -  1 | -  9 | -  0.1 | IC |
| C40975_g3_i1_1  (aa30-189) | β-tubulin | Q64FW3_9CNID  (aa1-160) | 96.9 | 1.3e-68 | S  H | 3  2 | 8  17 | 0.2  0.1 | IC |
| C40975_g4_i2_1  (aa1-276)  C40975_g4_i1_1 | β-tubulin; shares 2 peptides with C40975_g2_i3_1 | Q26165_PATVU  (aa166-440) | 98.2 | 3.5e-111 | S  H | 7  7 | 27  40 | 0.2  0.1 |  |
| C41002_g1_i1_6  (aa5-661)  C41002_g1_i5_4  C41002_g1_i4_5 | Uncharacterized; domain: VPS10, TMH (aa632-654,701-719); pI 5.9 | I1G6B6_AMPQE  (aa93-752) | 36.7 | 4.6e-70 | S  H | -  3 | -  9 | -  <0.1 | PM |
| C41069_g1_i1_3  (aa13-780)  C41069_g1_i2_3 | Uncharacterized; SSP (aa27/28), TMH (aa729-751,792-814,840-862,869-886,891-910,946-965); 10%L; pI 6.9 | R7TP97_CAPTE | 51.0 | 5.6e-180 | S  H | 3  5 | 23  44 | <0.1  <0.1 | ? |
| C41075_g1_i4_4  C41075_g1_i2_4 | β-actin; shares 4 peptides with c14026_g1_i1_3 and 1 with c3544_g1_i1 and c21396_g1_i1_4 | V5YU14_PATPE | 98.4 | 5.9e-160 | S  H | 13  13 | 115  127 | 1.9  0.8 | IC |
| C41117_g3_i2_4  (aa1-127)  C41117_g3_i3_5  C41117_g3_i1_5  C41117_g3_i4_6 | Histone H4 | K7LZT4_SOYBN  (aa41-160) | 86.8 | 2.1e-39 | S  H | 7  9 | 48  45 | 0.8  0.4 | IC |
| C41202_g1_i2_5  (aa42-492)  C41202_g1_i4_5 | α-tubulin; share 3 peptides with C41202_g1_i3_5 | Q27122_URECA  (aa1-451) | 96.9 | 5.6e-197 | S  H | -  1 | -  9 | <0.1 | IC |
| C41202_g1_i3_5 | α-tubulin; shares 3 peptides with C41202_g1_i2_5 | Q27122_URECA  (aa1-451) | 97.1 | 1.5e-198 | S  H | 5  5 | 19  21 | 0.1  0.1 | IC |
| C41224_g1_i2_6  (aa3-380)  C41224_g1_i1_5  C41224_g1_i4_5  C41224_g1_i3_6 | Uncharacterized; domain: VWA (aa58-238); DI ~aa375-400; 11% S; pI 4.3 | W5NB33_LEPOC  (aa199-587) | 34.5 | 1.5e-44 | S  H | -  2 | -  4 | -  <0.1 | ? |
| C41264_g1_i4_5  (aa1-523)  C41264_g1_i1_4 | Uncharacterized; domain: haem_peroxidase (aa16-498); TMH (aa550-572); 11% L; pI 7.8; shares 1 peptides with C41264_g1_i3_4 | I1FXG8_AMPQE  (aa2208-2733) | 44.3 | 5.6e-102 | S  H | 3  9 | 3  36 | <0.1  0.1 | ? |
| C41264_g1_i3_4  (aa1-285) | Uncharacterized; domain: haem_peroxidase (aa1-282); shares 1 peptide with C41264_g1_i4_5 | I1FXG8_AMPQE  (aa2448-2733) | 45.8 | 4.5e-58 | H  S | -  2 | -  4 | <0.01 |  |
| C41299_g2_i1_6  (aa67-333) | Uncharacterized; domain: fibrinogen_ α,β,γ_C_term_glob (aa49-171); pI 6.8; TMH (aa354-376,408-430) | I1F7N6_AMPQE  (aa268-533) | 40.5 | 8.4e-43 | S  H | 1  3 | 2  9 | <0.1  0.1 | ? |
| C41305_g2_i3_6  (aa1-762) | Uncharacterized/similar to SCUBE; domains: IGFBP, CUB, SSP (aa21/22), TMHs | H3CFP6_TETNG  (aa140-918) | 21.0 | 3.8e-11 | S  H | -  3 | -  16 | -  <0.1 | EC |
| C41377_g2_i1_1 | Uncharacterized; domains: IG (58-137), DUF4440 (172-278); shares 4 peptides with c41377_g2_i2_1; TMH (aa367-389); pI 8.5 |  |  |  | S  H | 2  7 | 7  43 | 0.1  0.2 | ? |
| C41377_g2_i2_1 | Uncharacterized; shares 4 peptides with c41377_g2_i1_1; domain: IG (aa58-136); TMH (aa350-572,392-414), 10% L; pI 9.1 |  |  |  | S  H | 4  3 | 11  30 | 0.1  0.1 |  |
| C41391_g2_i4_1  (aa4-316)  (aa320-678) | Similar to kielin/chordin-like protein; domains: VWD, uncharacterized_cys_ rich, 1 peptide  Uncharacterized;domains: trypsin_inhibitor_like_cys _rich, TMH (aa557-579,624-646); 3 peptides | L9JG99_TUPCH  (aa1166-1479) | 32.5 | 3.4e-29 | S  H | -  4 | -  16 | -  <0.1 | EC |
| C41414_g3_i1_2  C41414_g4_i1_3 | Similar to peroxiredoxin_like; domains: LRR (aa43-246), IG (aa150-241); TMH (aa10-32,275-297); 13% L, 10% S; pI 8.4 | M3WYC8_FELCA  (aa33-235) | 32.9 | 2.7e-18 | S  H | 1  1 | 16  31 | 0.1  0.1 | ? |
| C41463_g1_i1_6  (aa5-482) | Similar to core protein of aggregation factor (fragment); domain: Na-Ca exchanger/integrin_β4 (aa65-166); 10% V; pI 4.2 | Q94621_CLAPR  (aa560-1055) | 35.5 | 8.6e-37 | S  H | -  2 | -  3 | -  <0.1 | PM |
| C41463_g2_i1_6  (aa1-108) | Uncharacterized; pI 7.2 | I1G7C5_AMPQE  (aa28-137) | 39.3 | 2.6e-15 | S  H | -  2 | -  7 | -  0.1 | ? |
| C41502_g1_i1_2  (aa1-548)  C7365_g1_i1_6  (aa10-67) | Uncharacterized; domains: EGF, DOMON; 10% S; pI 4.5; EP-repeats aa198-226)  Uncharacterized;domain: DOMON (aa22-73); 10% E, 14% G, 10% S; pI 4.8 | B3SAZ3_TRIAD  (aa122-668)  I1FFF9_AMPQE  (aa143-700)  I1FFF9_AMPQE  (aa725-784) | 37.3  42.5  46.7 | 5.0e-44  4.7e-31  5.7e-5 | S  H  S  H | -  1  -  2 | -  9  -  5 | -  <0.1  -  0.1 | ? |
| C41515_g2_i1_5  (aa50-291) | Uncharacterized; domain: tropomyosin (aa55-290); 12% L, pI 6.6 | I1F8D0_AMPQE | 38.7 | 7.8e-10 | S  H | -  3 | -  3 | <0.01 |  |
| C41584_g1_i4_5  (114-300)  C41584_g1_i2_5  C41584_g1_i5_5 | Uncharacterized/similar to cytochrome c oxidase subunit 3; TMH (aa81-100,304-326,330-352); pI 6.6; 10% L, 13% S; shares 1 peptide with C41584_g1_i8_4 | G8HT99_ACRMI  (aa58-257) | 29.0 | 1.7e-5 | S  H | -  4 | -  28 | -  0.2 | IC |
| C41584_g1_i8_4  (aa180-347) | Uncharacterized; 10% I, 12% L; pI 6.9; TMH (aa7-29) ; shares 1 peptide with C41584_g1_i4_5 | G8HT99_ACRMI  (aa82-257) | 30.1 | 2.0e-10 | H  S | 2  3 | 9  18 | 0.1  0.1 | IC |
| C41614_g1_i6_6  (aa136-328)  C41614_g1_i2_5 | Uncharacterized; domains: fibrinogen_ α,β,γ_C_term_glob (aa122-334); TMH (aa33-55,364-381,391-410); 10% I; pI4.7 | I1FTS1_AMPQE  (aa71-263) | 47.2 | 3.2e-33 | S  H | -  2 | -  5 | -  0.1 | EC |
| C41619_g1_i10_5 | Similar to gelsolin; domains: villin/gelsolin; 10% K, pI 8.5 | I1FBN9_AMPQE | 67.8 | 3.1e-108 | S  H | -  3 | -  3 | -  <0.01 | IC |
| C41679_g3_i1_3 | Similar to Protein kinase C-binding protein NELL1/2; domains:IGFBP/EGF; 13% C; pI 5.3 | I1GDQ3_AMPQE  (aa310-861) | 34.0 | 2.1e-43 | S  H | 1  6 | 8  21 | 0.1  0.1 | EC |
| C41693_g1_i7_5  C41693_g1_i3_6 | Uncharacterized; domain: fibrinogen_ α,β,γ_C_term_glob (aa12-80); pI 5.6 | I1EQR1_AMPQE  (aa112-375) | 40.9 | 5.1e-3 | S  H | 1  3 | 19  46 | 0.1  0.2 | EC |
| C41727_g3_i2_1  (aa42-415)  C41727_g3_i3_1 | Uncharacterized/lysosomal lipase; domain: A/B_hydrolase; 10% L; pI 6.0 TMH (aa20-38) | G3P6H1_GASAC  (aa34-403) | 45.9 | 4.1e-76 | S  H | 2  5 | 15  33 | 0.1  0.1 | IC |
| C41731_g1_i3_5 | Uncharacterized; 10% I, 12% L, 10% S; pI 9.2; TMH (aa5-27,573-595, 616-638,658-680,700-722,742-764) |  |  |  | S  H | 1  9 | 13  59 | 0.1  0.2 | ? |
| C41884_g1_i6_4  (aa3-403)  C41884_g1_i2_4  C41884_g1_i1_6 | Uncharacterized/similar to prolyl 3-hydroxylase 2; domains: tetratrico-peptide_like_helical (aa161-194), IG/transglutaminase_C_term (aa548-636)  11%L; pI 8.8 | V4ALP2_LOTGI  (aa90-481) | 32.7 | 1.6e-41 | S  H | 2  4 | 6  23 | <0.1  <0.1 | IC |
| C41889_g3_i8_5  C41889_g3_i4_5 | Similar to thioester-containing protein; domains: A2M; 10% S; pI 6.0 | D7R6D5_BIOGL  (aa326-1356) | 25.9 | 9.6e-52 | S  H | 2  8 | 5  50 | <0.1  <0.1 | EC |
| C53357_g1_i1_2  (aa1-85) | Proteasome subunit alpha type-7-B | PSA7B_XENLA  (aa136-220) | 98.8 | 1.0e-33 | S  H | -  2 | -  5 | -  0.1 | IC |
| C53634_g1_i1_3 | Uncharacterized; 18% D, 12% E, 13% I, 10% V; pI 3.5; 68aa; Only one possible peptide? |  |  |  | S  H | 1  1 | 100  113 | 8.2  5.6 | ? |
| C54677_g1_i1_2 | Ubiquitin | Q5QBF8_CULSO | 99.4 | 1.5e-65 | S  H | 6  5 | 198  127 | 2.7  1.3 | IC,EC |
| C58706_g1_i1_2 | Uncharacterized collagen (fragment); 35% G, 14% P; pI 9.4 |  |  |  | S  H | 1  1 | 4  15 | <0.1  0.1 | EC |
| C64227_g1_i1_3 | Uncharacterized; domain: fibrinogen _α,β,γ_C_term_glob (aa1-82); 10% L; pI 8.2; TMH (aa153-172) |  |  |  | S  H | 3  3 | 39  74 | 1.1  0.7 | ? |
| C65284_g1_i1_4  C6913_g1_i1_1 | Heat shock cognate protein 70 (HSPA8); pI 6.5; shares 2 peptides with c94913_g1_i1_1 and 1 with c35750_g1_i2_1 | Q7ZTK6_XENLA  (aa159-372)  (aa362-586) | 100.0  100.0 | 5.1e-81  2.8e-79 | S  H  S  H | 4  7  1  2 | 20  19  2  6 | 0.2  0.1  <0.1  <0.1 | IC,EC |
| C7761_g1_i1_1  (aa1-181) | Similar to astrosclerin; domain: α-CA (aa1-160); 11% L; pI 5.6; shares 1 peptide with c94004_g1_i1_2 | A6YCJ0_9METZ  (aa109-290) | 46.2 | 4.9e-29 | S  H | 2  4 | 15  23 | 51.6  31.9 | ? |
| C77644_g1_i1_3  (aa1-55) | Uncharacterized; domain: fibrinogen_α,β,γ_C_term_glob, subdomain_2 (aa1-55), TMH (aa89-111); 10% I, 10% L; pI 6.0 | H2Y8G7_CIOSA  (aa184-240) | 45.6 | 3.5e-4 | S  H | -  3 | -  36 | -  2.9 | EC |
| C80184_g1_i1_1  (aa1-130) | Annexin (A2) | Q92128_XENLA  (aa42-171) | 99.2 | 8.8e-50 | S  H | -  2 | -  6 | -  0.1 | IC,EC |
| C80614_g1_i1_3 | Similar to peroxidasin; domain: haem_peroxidase; 10% R, 13% L; pI 8.8 | V6GWB1_9CNID  (aa692-773) | 54.9 | 4.6e-18 | S  H | 2  2 | 9  33 | 0.3  0.6 | ? |
| C91905_g1_i1_2  (aa1-131) | Similar to Ig domain protein group 1 domain protein; 10% A; pI 4.8 | G0IYW5_CYCMS  (aa264-388) | 36.1 | 2.1e-8 | S  H | -  2 | -  9 | -  <0.1 | ? |
| C94004_g1_i1_2 | Uncharacterized; shares 1 peptide with C7761_g1_i1_1 (astrosclerin) |  |  |  | S  H | -  2 | -  3 | -  4.1 |  |
| C94913_g1_i1_1  (aa23-676) | Heat shock cognate 70 kDa protein; shares 2 peptides with c65248_g1_i1_4 and 1 with c6913_g1_i1_1; pI 5.5; TMH (aa681-703) | B5X3U6_SALSA  (aa5-663) | 83.0 | 4.6e-171 | S  H | 4  5 | 27  28 | 0.1  0.1 | IC,EC |
| C95078_g1_i1_2 | Uncharacterized; domain: IG (aa1-110); 12% L, 10% S, 12% V; pI 8.2; TMH (aa47-69) |  |  |  | S  H | 1  1 | 14  26 | 0.4  0.4 | ? |
| C97612_g1_i1_1 | Similar to DBH-like monooxygenase protein 2-like protein; domain: DOMON (aa1-91); 11% D, 11% S; pI 4.2 | K1QE34_CRAGI  (aa373-466) | 41.1 | 8.5e-11 | S  H | 1  1 | 5  10 | 0.4  0.5 | ? |
| C99840_g1_i1_1 | Uncharacterized; 10% G, 11% I, 11% V; pI 5.2; TMH (aa236-258,273-290) |  |  |  | S  H | 3  6 | 23  114 | 0.8  5.8 | ? |
|  |  |  |  |  |  |  |  |  |  |

**^1^**, rounded to one decimal place. **^2^**, derived from similarity to other proteins, predicted domains, predicted secretion signal sequence, or predicted transmembrane segments. IC, intracellular; EC, extracellular; PM, plasma membrane. TMH, predicted transmembrane helix. DI, predicted disordered structure.
