# Supplementary material for: The Skeleton Forming Proteome of an Early Branching Metazoan: A Molecular Survey of the Biomineralization Components Employed by the Coralline Sponge Vaceletia Sp
Source: PLoS One. 2015 Nov 4;10(11):e0140100. doi: 10.1371/journal.pone.0140100 (PMC4633127; doi:10.1371/journal.pone.0140100)
Supplement: S2 Table — (PDF) [file pone.0140100.s008.pdf]

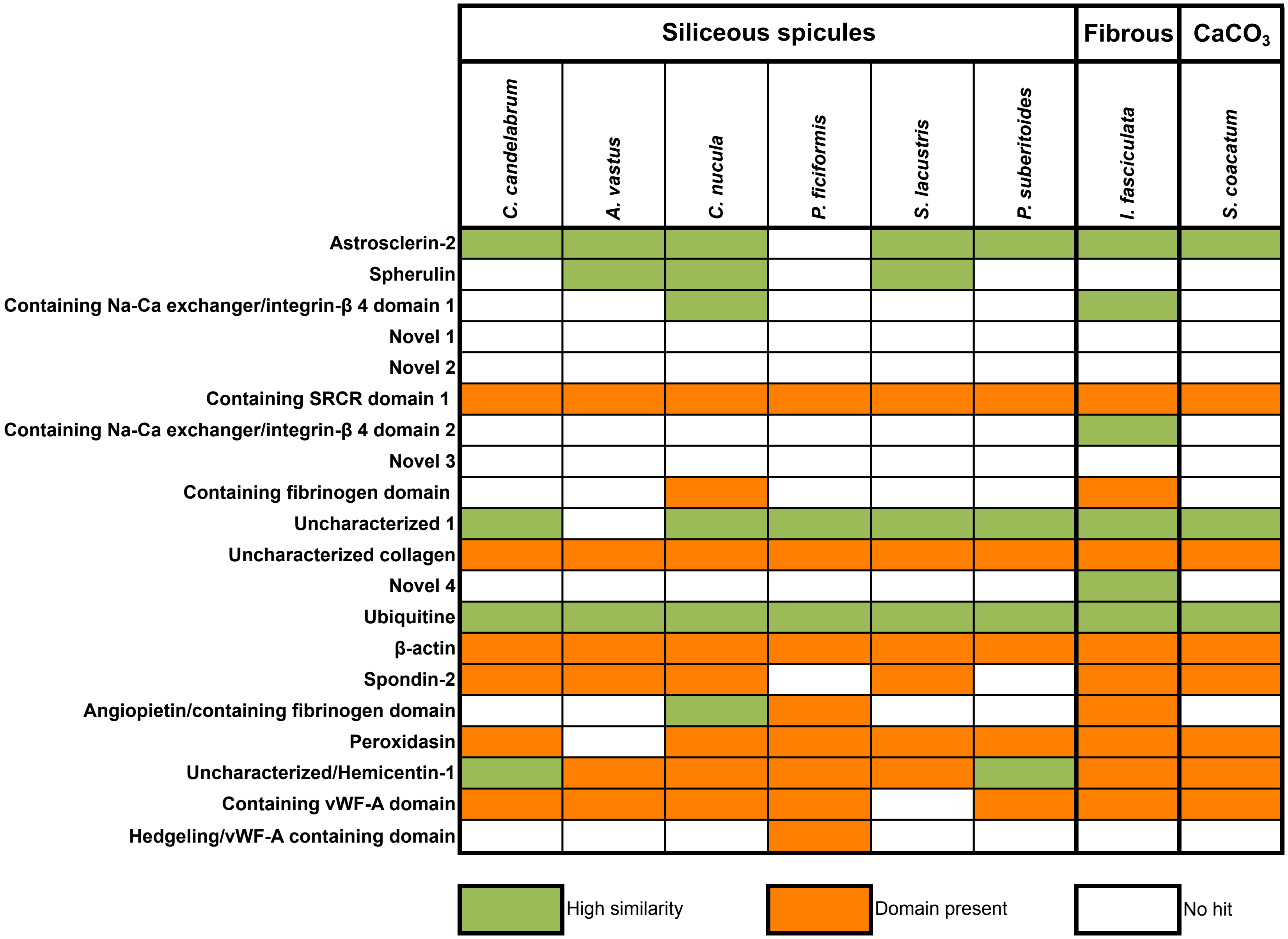
Siliceous spicules
Fibrous
$CaCO_3$
C. candelabrum
A. vastus
C. nucula
P. ficiformis
S. lacustris
P. suberitoides
I. fasciculata
S. coacatum
Astrosclerin-2
Spherulin
Containing Na-Ca exchanger/integrin-β 4 domain 1
Novel 1
Novel 2
Containing SRCR domain 1
Containing Na-Ca exchanger/integrin-β 4 domain 2
Novel 3
Containing fibrinogen domain
Uncharacterized 1
Uncharacterized collagen
Novel 4
Ubiquitine
β-actin
Spondin-2
Angiopietin/containing fibrinogen domain
Peroxidasin
Uncharacterized/Hemicentin-1
Containing vWF-A domain
Hedgeling/vWF-A containing domain
High similarity
Domain present
No hit

The 20 contigs of *V.sp* that were used in the comparison analyses against the eight sponge transcriptomes

Astrosclerin-2	C7761_g1_i1_1
Spherulin	C32545_g1_i1_1/ C32545_g1_i2_1
Containing Na Ca exchanger/integrin-β 4 domain 1	C38723_g1_i1_3
Novel 1	C99840_g1_i1_1
Novel 2	C53634_g1_i1_3
Containing SRCR domain 1	C36962_g2_i1_6
Containing Na Ca exchanger/integrin-β 4 domain 2	C23124_g1_i2_3/ C23124_g1_i1_3
Novel 3	C94004_g1_i1_2
Containing fibrinogen domain	C77644_g1_i1_3
Uncharacterized 1	C32287_g1_i1_1
Uncharacterized collagen	C29357_g1_i1_2
Novel 4	C22072_g1_i1_3
Ubiquitine	C54677_g1_i1_2
β-actin	C3544_g1_i1_1
Spondin-2	C37591_g1_i3_5
Angiopietin/containing fibrinogen domain	C1963_g1_i2_2
Peroxidasin	C80614_g1_i1_3
Uncharacterized/Hemicentin-1	C40964_g7_i1_1/ C40964_g7_i2_1/ C40964_g7 _i4_2
Containing vWF-A domain	C40249_g1_i3_3/ C40249_g1_ i2_3/ C40249_g1_i1_3
Hedgeling/vWF-A containing domain	C100960_g1_i1_4
